# Regeneration leads to global tissue rejuvenation in aging sexual planarians

**DOI:** 10.1038/s43587-025-00847-9

**Published:** 2025-04-03

**Authors:** Xiaoting Dai, Xinghua Li, Alexander Tyshkovskiy, Cassandra Zuckerman, Nan Cheng, Peter Lin, David Paris, Saad Qureshi, Leonid Kruglyak, Xiaoming Mao, Jayakrishnan Nandakumar, Vadim N. Gladyshev, Scott Pletcher, Jacob Sobota, Longhua Guo

**Affiliations:** 1 Department of Molecular & Integrative Physiology, University of Michigan, Ann Arbor, MI USA; 2 Institute of Gerontology, Geriatrics Center, University of Michigan, Ann Arbor, MI USA; 3 Division of Genetics, Department of Medicine, Brigham and Women’s Hospital, Harvard Medical School, Boston, MA USA; 4 Department of Molecular, Cellular, and Developmental Biology, University of Michigan, Ann Arbor, MI USA; 5 Department of Physics, University of Michigan, Ann Arbor, MI USA; 6 Department of Human Genetics, Department of Biological Chemistry, Howard Hughes Medical Institute, University of California, Los Angeles, Los Angeles, CA USA; 7 Broad Institute, Cambridge, MA USA

**Keywords:** Senescence, Ageing, Ageing, Ageing

## Abstract

The possibility of reversing the adverse impacts of aging could significantly reduce age-related diseases and improve quality of life in older populations. Here we report that the sexual lineage of the planarian *Schmidtea mediterranea* exhibits physiological decline within 18 months of birth, including altered tissue architecture, impaired fertility and motility, and increased oxidative stress. Single-cell profiling of young and older planarian heads uncovered loss of neurons and muscle, increase of glia, and revealed minimal changes in somatic pluripotent stem cells, along with molecular signatures of aging across tissues. Remarkably, amputation followed by regeneration of lost tissues in older planarians led to reversal of these age-associated changes in tissues both proximal and distal to the injury at physiological, cellular and molecular levels. Our work suggests mechanisms of rejuvenation in both new and old tissues concurring with planarian regeneration, which may provide valuable insights for antiaging interventions.

## Abstract (web summary)

The freshwater planarian *S. mediterranea* is an important model organism for regeneration research but its use in aging research is little explored and *S. mediterranea* is commonly referred to as immortal. Here Dai et al. report age-associated physiological and molecular changes in the sexual lineage of *S. mediterranea*, and global reversal of such changes after regeneration.

## Main

Adult stem cells (ASCs) undergo self-renewal and differentiation to replace cells lost to wear and tear. In old age, the regenerative capacities of ASCs diminish^1–5^, and this phenomenon is one of the major causes of frailty and age-related diseases^1^. Humans begin losing skeletal muscle in their 40s, and by the age of 80 years, up to 50% of muscle mass may be lost, contributing to impaired function and disability^6,7^. Similarly, neuronal loss occurs in the human hippocampus, beginning at age 13 and continuing into the 80s^8^. Age-associated loss of neurons is even more devastating in the context of neurodegenerative diseases, such as Alzheimer’s disease^9,10^. A method for rejuvenating aged ASCs and differentiated tissues would revolutionize medicine and significantly aid efforts to combat aging and age-related disease.

Several interventions have been shown to rejuvenate old cells in multiple tissues^2,4^. These include heterochronic parabiosis^11^, exercise^12–14^, caloric restriction^15^ and reprogramming^2,4,16^. However, the aged hematopoietic stem cell system, which is critical to mammalian healthspan and lifespan, remains refractory to these interventions^2,17^ and reprogramming reagents have to be delivered in a tissue-specific fashion^16^. Hence, while rejuvenation research has shown great promise in restoring functions in multiple tissues, global rejuvenation of all tissues or age reversal of whole-animal physiology remains elusive.

Long-lived species provide unique opportunities to uncover naturally evolved mechanisms for the extension of healthspan and lifespan^18^. Freshwater planarians are commonly referred to as immortal due to their extremely long lifespan^19–23^ and unique tissue regeneration capabilities^24,25^. It was reported that telomeres shorten^26^, eyes change and viable progeny decline^27^ in older planarians. Whether planarians experience aging and show a typical age-dependent decline in physiological, cellular and molecular functions has not been systematically examined, in part because of the challenges inherent in measuring lifespan in a long-lived animal, or even defining age in asexual planarians that undergo a vegetative mode of reproduction^28^. Inbred lines of the sexual lineage of *S. mediterranea* have been established to study genetic variations and chromosome biology^29,30^. This resource provides a unique opportunity to examine aging in this long-lived model system and disentangle genetic control from environmental effects.

Here, we examine aging in the sexual lineage of *S. mediterranea*. To use this model for aging research, we define chronological age as time since fertilization, thus overcoming the challenges involved in lineages that rely on vegetative reproduction. As in the more traditional short-lived model systems (mice, fish, nematodes and fruit flies), *S. mediterranea* exhibits signs of aging at multiple levels—molecular, cellular and physiological. However, we found that amputation and regeneration trigger global rejuvenation that reverses the multilevel effects of aging.

## Results

### Amputations lead to reduction of EEP phenotypes

**Fig. 1:**
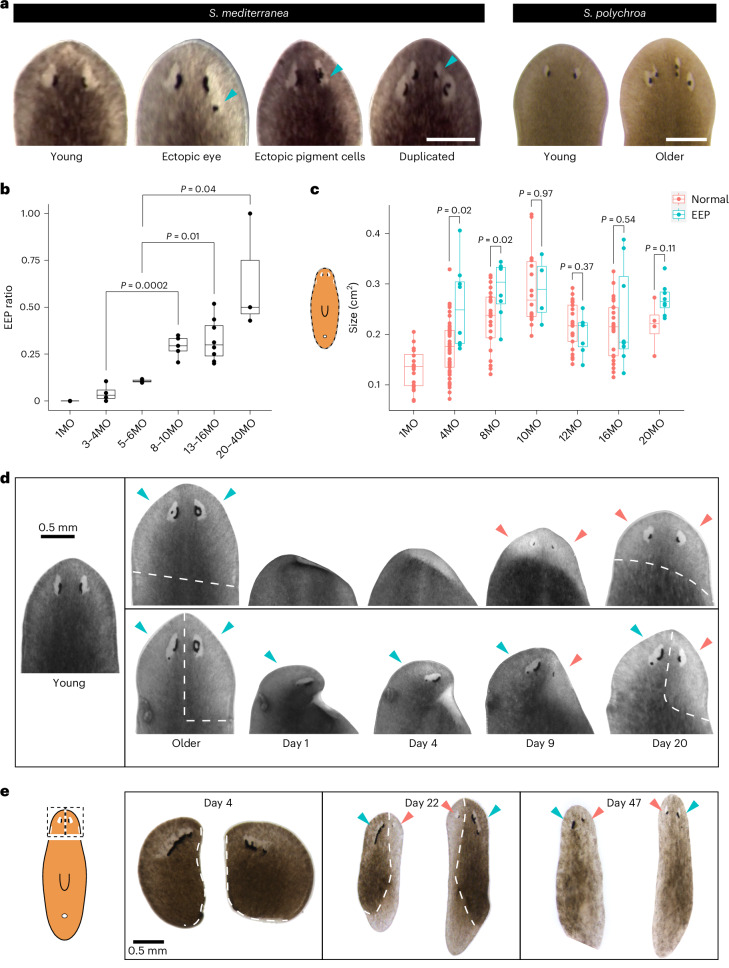
Aging and rejuvenation of planarian eyes. **a**, Morphological changes in the eyes of *S. mediterranea* and *S. polychroa*, from left to right. Cyan arrowheads indicate ectopic eyes, ectopic eye pigment cells and duplicated eyes (EEP). Scale bars, 0.5 mm. **b**, The ratio of worms with EEP phenotypes as a function of age. Each data point represents an independent container of worms with *n* ≥ 4 individuals and 744 individuals were sampled. Two-sided standard two-sample *t*-test. MO, months. **c**, Whole-body size changes with age in worms with and without EEP. Each data point represents an individual animal. *n* ≥ 12 for each age condition and a total of 200 individuals were included. Two-sided Welch’s *t*-test. In the box plots (**b** and **c**), the center is the median, the height of the box is given by the interquartile range (IQR), the whiskers extend up to 1.5 times the IQR, and the minima and maxima are the observed minima and maxima. **d**, Whole-head regeneration (top, *n* = 15) and half-head regeneration (bottom, *n* = 10) of worms with EEP. **e**, Regeneration of a half head with EEP into a new worm (*n* = 5). Dashed line indicates the amputation plane. Cyan arrowheads indicate old eyes; red arrowheads indicate new eyes. Scale bar, 0.5 mm. Source data

To study aging in planaria, we turned to *S. mediterranea* strains that proliferate via sexual reproduction and defined zygotes as age zero. We found that most of these strains, including both wild isolates of unknown ages from the islands of Corsica and Sardinia and lines bred in captivity, develop visible morphological changes in the eyes between 6 months (6MO) and 5 years of age. The most prominent phenotype is the development of ectopic eyes or pigment (EEP) (Fig. 1a). This eye phenotype is also observed in isolates of a closely related species, *Schmidtea polychroa*, directly captured in the wild from Sardinia (Fig. 1a; 2 of 10 with abnormal eyes).

**Extended Data Fig. 1:**
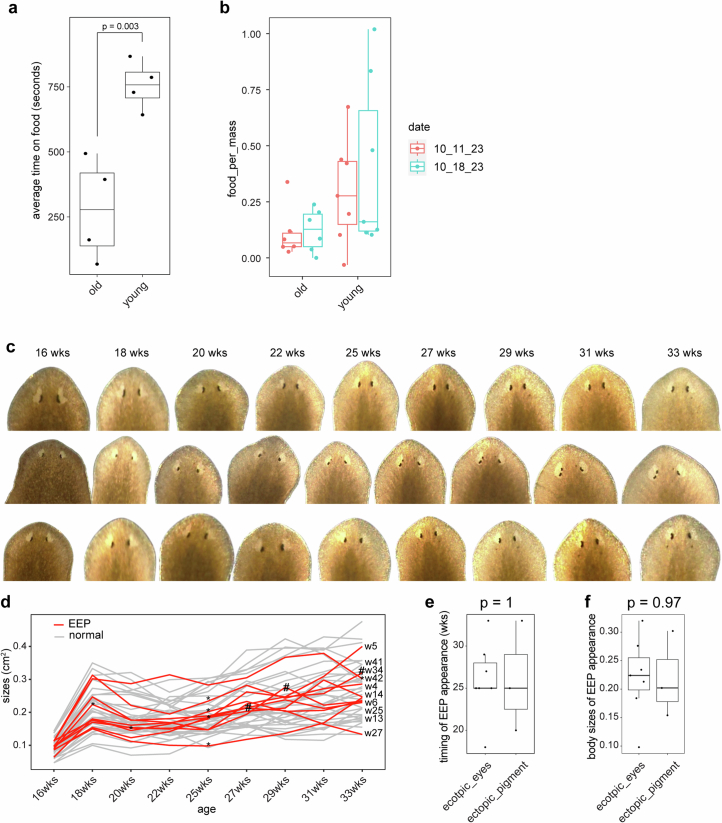
Age associated changes in feeding behavior and eye morphology. **(a**) Average time spent on food in four independent feeding sessions for young (8MO) and old (18MO) planarians. Each dot represents the average time from 8 animals that were examined independently in one feeding session. Two-sided Welch Two Sample t-test. (**b**) The amount of liver ingested per feeding session, normalized by the weight of the animal (mg/mg). Each dot represents one animal. Young, 7MO: 7 animals. Old, 12MO: 6 animals. Red and cyan represent two feeding sessions of the same animals. (**c**) Development of EEP phenotypes over time in three animals from 4MO to 8MO. From top to bottom: ectopic eye, ectopic eye pigment, duplicated eyes. wks = weeks-old (**d**) Body size changes over time in 44 individually housed animals, from 4MO to 8MO. Red lines: animals developed early signs of EEP. Grey lines: animals with normal eyes. *: the first time point when EEP was visible. #: the first time point when EEP was visible, and the animal was at its largest size. w4, w5, w6…w42: worm IDs, corresponding to eye phenotypes in source data. (**e-f**) No significant differences in the timing of EEP appearance (**e**) or body sizes of EEP appearance (**f**) between ectopic eyes and ectopic pigment cells. Each data point represents an individual with EEP, *n* = 10 individuals were examined. Welch two sample t-test. (**a-b, e-f**) The boxplots show the median (center), IQR (box) and whiskers extending to 1.5 × IQR. Source data

To comprehensively determine if development of EEP is dependent on age, we examined 761 planarians from the age of 1MO to 40MO. We found no EEP in 1MO planarians. From 3MO to 40MO, the percentage of planarians with EEP steadily increased from 4% to 64% (Fig. 1b). As planarians continue to grow bigger in size after 1MO, we measured the sizes of 200 planarians from 1MO to 20MO and recorded their eye phenotypes. From 1MO to 10MO, planarians with normal eyes steadily increased in size (Fig. 1c). At 4MO and 8MO, planarians did not develop EEP until they reached much bigger sizes (two-sided unpaired two-sample *t*-test; *P* = 0.02 at both ages between normal eyes and EEP). However, large size alone is not sufficient to cause the development of EEP. When we measured planarians that had reached their maximal sizes (10MO or older), we saw that worms with normal eyes were no different in size from worms with EEP. Surprisingly, we found planarians from 12MO to 20MO are relatively smaller than their maximal sizes, which is likely due to reduced food intake in older ages (Extended Data Fig. 1a,b). We used linear regression in R to model the contributions of sizes and ages to the development of EEP. Both age and size are strong predictors of EEP (*P* = 0.007 and 0.001 for age and size, respectively). As the experimental data showed that EEP ratios continued to rise in older ages (Fig. 1b) when EEP animals were no longer significantly different from normal animals in size (Fig. 1c), we conclude that EEP is an age-dependent phenotype, with large body size being a prerequisite condition for developing this phenotype.

To further support the above conclusion, we housed 44 planarians in solitude, and tracked EEP phenotypes and changes in body size for every individual from 4MO to 8MO (Extended Data Fig. 1c,d). At 8MO, 10 of 44 animals developed early stages of EEP phenotypes, with 3 of them as ectopic pigment and 7 of them as ectopic eyes. At the time of EEP cell appearance, 7 of 10 animals were not at their largest body size (Extended Data Fig. 1d), providing further support that aging contributes to the development of EEP. Age and size of animals were not significantly different between ectopic eyes and ectopic pigment cells when examined at the time of EEP appearance (Extended Data Fig. 1d–f).

We noticed that amputating the heads of old planarians exhibiting EEP led to the generation of new heads lacking this phenotype (Fig. 1d). We wondered whether the amputation and regeneration process had ameliorated tissue aging phenotypes. Therefore, we experimented with 32MO individuals with EEP. We either removed the whole head to examine whether the remaining body would regenerate a head with two normal eyes (Fig. 1d), or removed half of the head to examine whether the newly regenerated half of the head would have a normal eye (Fig. 1d). In both experiments, amputation and regeneration produced normal eyes. To determine if the EEP eye that did not require regeneration (Fig. 1d) can be corrected, we carried out more extensive amputations and followed the changes of EEP throughout regeneration (Fig. 1e). We found the EEP phenotype can be corrected 47 days post amputation (47Dpa) in the head fragments, suggesting more extensive tissue remodeling is needed to correct EEP structures.

**Fig. 2:**
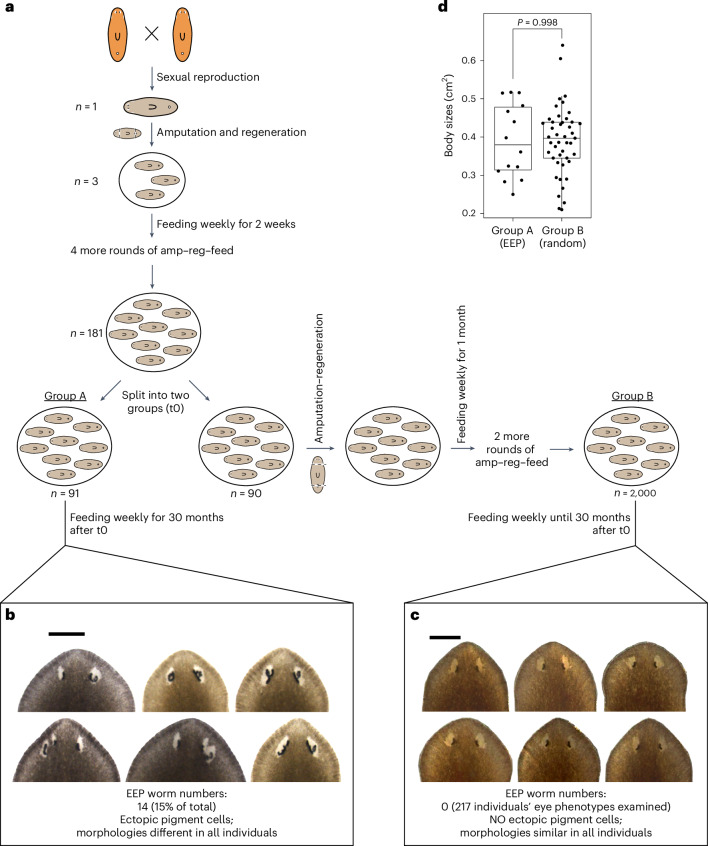
Repeated amputation and regeneration maintain youthful heads. **a**, Strategy to create the aging cohort (group A) and regenerated cohort (group B) with the same genetic background and chronological age. amp, amputation; reg, regeneration; feed, feeding. **b**, Representative images of six clones with heterogeneous EEP phenotypes from group A. **c**, Representative images of six clones with homogeneous young eyes from group B. **d**, Whole-body size comparison between EEP worms in group A and randomly sampled 45 worms in group B. Each data point represents an individual animal (*n* = 14 and 45 in group A and B, respectively). Two-sided Welch’s *t*-test. The box plot shows the median (center), IQR (box), whiskers extending to 1.5 times the IQR and the observed minima and maxima (minima and maxima). Scale bars, 0.5 mm. Source data

To further characterize this observed effect of amputation on EEP structures and to determine whether repeated rounds of amputation, regeneration and feeding (amp–reg–feed) lead to delayed aging, we compared the eye aging phenotypes of two cohorts of clones that were created from a single individual and were both chronologically 36MO. The ancestral individual was obtained by mating a pair of adults (Fig. 2a). Repeated amp–reg–feed of this single individual resulted in a cohort of 181 clones. We divided this cohort into a normal aging group (group A), which contained 91 clones and was maintained on a weekly feeding schedule for 30 additional months, and a repeated-regeneration group (group B), which initially contained 90 clones but underwent three additional rounds of amp–reg–feed over the same 30-month periods. For repeated regeneration, regenerated fragments were mixed and fed once a week until they reached sexual maturity, at which point we initiated the next round of amp–reg–feed. At the end of the experiment, group B contained ~2,000 clones (Fig. 2a). We observed that 14 of the 91 individuals in group A (15%) had developed EEP, with sufficient heterogeneity in the phenotypes to distinguish one individual from another (Fig. 2b). If the two groups had the same aging rates, we would have expected 15% of the clones in group B to develop EEP. Contrary to this expectation, a random sampling of 217 individuals from group B did not find any individuals exhibiting EEP (0%, chi-squared test, d.f. = 1, *P* < 0.001; Fig. 2c). All individuals in group B showed a uniform eye morphology. Body sizes between group A-EEP worms and group B worms were not significantly different (Fig. 2d). These experiments suggest that repeated amp–reg–feed cycles can potentially maintain a youthful state and delay the occurrence of the aging eye phenotypes.

### Effect of amputation on age-associated physiological changes

**Fig. 3:**
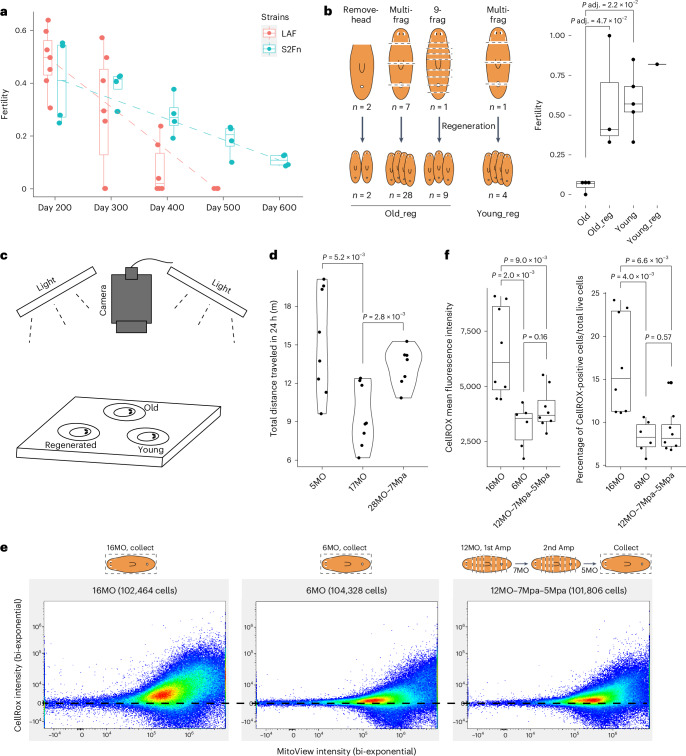
Aging and rejuvenation of fertility, motility and oxidative stress state. **a**, Tracking of fertility over time in two different inbred lines, LAF and S2Fn. Fertility was quantified as the percentage of hatched egg capsules to the total eggs produced every 100 days. Each data point represents a box of animals (*n* = 7 and 4 for LAF and S2Fn, respectively). The box plot shows the median (center), IQR (box), whiskers extending to 1.5 times the IQR and the observed minima and maxima (minima and maxima). **b**, Left: three strategies of amputation to produce regenerated worms, including removing the heads, cutting animals into random 3–4 fragments and cutting animals into 9 fragments. Right: fertility of the first month of reproduction, from young, young-regenerated, old and old-regenerated cohorts. Young: 4MO, 6MO, 7MO. Old: 12MO, 13MO, 14MO, 24MO. Old_reg: 18MO–3Mpa (9-frg), 21MO–3Mpa (remove-head), 24MO–5Mpa (multi-frg), representative data for each method. Each method was repeated at least three times. Young_reg: 4.5MO–4Mpa. One-way analysis of variance (ANOVA) with Tukey’s honestly significant difference (HSD) test. The box plot shows the median (center), IQR (box), whiskers extending to 1.5 times the IQR and the observed minima and maxima (minima and maxima). **c**, Camera, light and chamber setup for video recording in the stress–motility assay. **d**, Total movements measured as distance traveled in 24 h for 5MO, 17MO and 28MO–7Mpa animals. Each data point represents an individual worm. *n* ≥ 6 individuals for different experimental groups. Two-sided Welch’s *t*-test. **e**, Flow cytometry analysis of oxidative stress states determined by CellROX Green staining in 6MO (*n* = 6), 16MO (*n* = 8) and 12MO–7Mpa–5Mpa (*n* = 8) animals. Amp, amputation. Horizontal dashed lines are added to help compare the density and intensity of CellRox^high^ cell populations. **f**, Quantification of CellROX mean fluorescence intensity and percentage of CellROX-positive cells in each age group from **e**. Each data point represents an individual worm. Two-sided Welch’s *t*-test. The box plots show the median (center), IQR (box), whiskers extending to 1.5 times the IQR and the observed minima and maxima (minima and maxima). Source data

To determine if the observed amputation effect impacted other age-related phenotypes more globally, we examined additional physiological traits. A common age-related trait across phyla is reproductive senescence^31^. We measured fertility by tracking the percentage of egg capsules hatched every 100 days in two inbred lines, S2Fn and Los Angeles Fertile (LAF), over 2 years. We found that fertility declined continuously from ~40% at day 200 to ~10% at day 600 in line S2Fn (Fig. 3a). Planarians older than 2 years continued to produce egg capsules, at a lower number, and <10% hatched, suggesting that reproductive senescence had occurred. In addition, we found that line LAF reached reproductive senescence faster than line S2Fn (Fig. 3a). This suggests genetic regulations in the rate of reproductive aging.

We then asked if reproductive senescence can be reversed by regeneration. We collected fertility data for young, old and regenerated cohorts of animals from line LAF. Fertility in the old cohorts (12–24MO) was ~6% (Fig. 3b). Regeneration of the 18–24MO cohorts restored fertility to 58% (Fig. 3b), comparable to that of the 4–7MO cohorts, which had an average fertility of 59% (Fig. 3b). Removing heads or cutting the animal into 3–9 fragments all rescued fertility after regeneration (Fig. 3b). Hence, we conclude that regeneration reverses age-related reproductive senescence.

Impaired physiological performance (for example, motility) is another characteristic of old age in mice and humans^32,33^. To test whether older planarians exhibit impaired performance and, if so, whether regeneration can reverse it, we measured the total distance traveled by individual animals in 24 h under stressful conditions (Fig. 3c,d). We found that young (5MO) planarians were more active than older (17MO) planarians, traveling an average of six more meters in a day (Fig. 3d). The regenerated cohort (28MO with 7 months post amputation (7Mpa); Methods, ‘Nomenclature’) traveled an average of four more meters in a day than the 17MO individuals (Fig. 3d). This stress–motility assay suggests regeneration also reverses age-associated decline in physiological performance.

Increased free radical and oxidative stress is considered a hallmark of aging^34,35^. To determine if aging in planarians is associated with an increase in reactive oxygen species (ROS), we used CellROX Green dye to stain dissociated cells from 6MO and 16MO planarians. CellROX will turn from weakly fluorescent to bright green and stably binds to DNA upon oxidation by ROS, allowing for quantitative analysis with flow cytometry. We quantified the intensity of green fluorescence for more than 100,000 cells per animal and repeated this analysis in 6–8 animals per condition (Fig. 3e,f). We found that older planarians consistently showed much more oxidation by ROS (Fig. 3e,f). To examine if regeneration reverses ROS activity in older planarians, we used animals that were chronologically 24MO, with two rounds of amputation and regeneration (12MO–7Mpa–5Mpa; Methods, ‘Nomenclature’). The 12MO–7Mpa–5Mpa planarians had a remarkably similar cellular profile to 6MO planarians (Fig. 3e), with no statistically significant differences in the mean intensity (Fig. 3f). Notably, cells of 16MO planarians were more heterogeneous and dispersed with CellROX intensity, than cells of the 6MO or 12MO–7Mpa–5Mpa planarians. We conclude that regeneration reverses age-associated increase of ROS activity.

### Characterizing the ASC system

Most animals exhibit age-related decline in stem cell function, which reduces regenerative abilities^2^. A decline in stem cell function could underlie the physiological effects we observed in aging planaria. Yet, the continued ability to regenerate may reflect maintenance of stem cell function. To examine stem cell aging in planarians, we characterized the types of ASCs found in the sexual lineage of *S. mediterranea*.

**Fig. 4:**
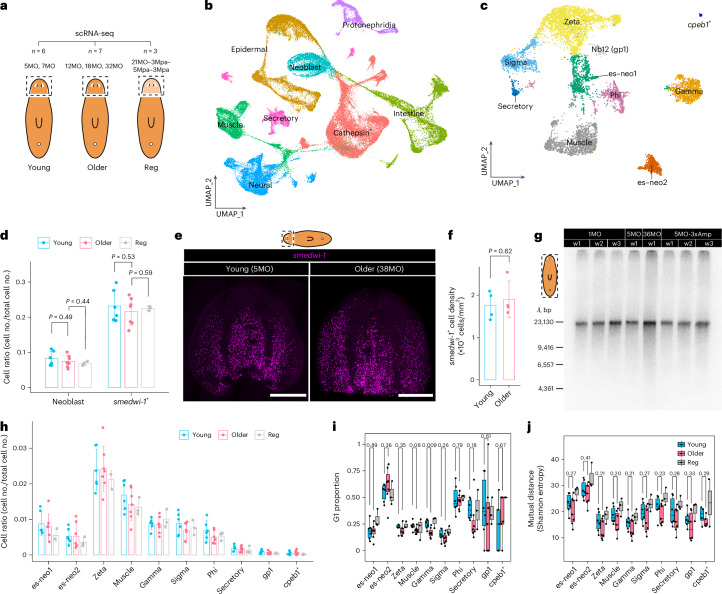
Single-cell profiling of the ASCs. **a**, Experimental outline for scRNA-seq on heads of young (5MO, *n* = 4; 7MO, *n* = 2), older (12MO, *n* = 1; 18MO, *n* = 5; 32MO, *n* = 1) and regenerated (21MO–3Mpa–5Mpa–3Mpa, *n* = 3) worms. Reg, regenerated. **b**, Uniform manifold approximation and projection (UMAP) visualization of eight major tissue types identified (*n* = 104,617 cells). **c**, Subclusters of sexual ASCs (neoblasts). **d**, Proportion of stem cells in different age groups in scRNA-seq data. The sample size for each experimental group is as indicated in **a**. The stem cells were quantified as the whole neoblast cluster or all *smedwi-1*^+^ cells. Two-sided Welch’s *t*-test. Error bars represent the mean ± s.d. **e**, HCR-FISH images of neoblast marker *smedwi-1* (magenta) in young and old planarian heads. *n* = 4 in each group. Scale bars, 500 µm. **f**, Quantification of *smedwi-1*^+^ cells in young and old planarian heads. Each data point represents an individual animal. Two-sided Welch’s *t*-test. Error bars represent the mean ± s.d. **g**, Telomere length of young (1MO, 5MO), old (36MO), and regenerated (5MO-3xAmp) planarians. One lane corresponds to one worm. **h**, Cell ratio of neoblast subtypes in three age groups from **a**. No significant differences between any two of these groups. The sample size for each group is indicated in **a**. Two-sided Welch’s *t*-test. Error bars represent the mean ± s.d. **i**, Fraction of stem cells at the G1 stage according to the predicted cell cycle state, derived from scRNA-seq data in **a**. The sample size for each experimental group is as indicated in **a**. Two-sided Welch’s *t*-test. **j**, Entropy (mutual distance) of neoblast subtypes. Each data point represents the mean entropy of a biological replicate derived from scRNA-seq data from **a**. The sample size for each experimental group is as indicated in **a**. Two-sided Welch’s *t*-test. The box plots in **i** and **j** show the median (center), the IQR (box) and whiskers extending to 1.5 times the IQR. Source data

**Extended Data Fig. 2:**
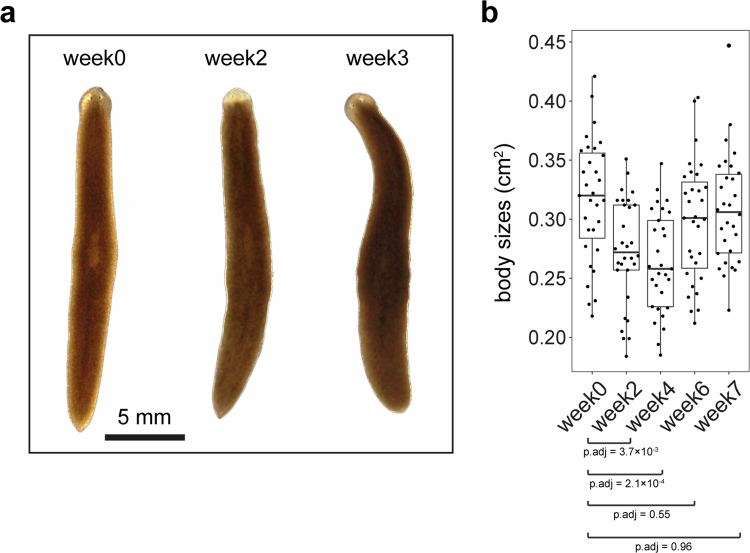
Head amputation and body size changes. **(a**) Representative images of intact (week0), 2-week post head removal (week2) and 3-week post head removal (week3) adult sexual planarians. (**b**) Body size changes after head removal. The data point represents individual worm. n = 31 individuals were sampled. One-Way ANOVA with Tukey’s HSD test. The boxplot shows the median (center), IQR (box) and whiskers extending to 1.5 × IQR. Source data

We used single-cell mRNA sequencing (scRNA-seq) to profile the cell types and states of 16 planarians at different ages and regeneration conditions (Fig. 4a). To improve the consistency of tissue sampling from different individuals, we used the posterior ends of the two auricles to mark the amputation sites and profiled cells from young (5–7MO), old (12–32MO) and regenerated (21MO–3Mpa–5Mpa–3Mpa) heads (Fig. 4a). Head removal by this procedure led to ~13% loss of body mass (Fig. 4a and Extended Data Fig. 2), minimizing the massive body remodeling that occurs in traditional amputation experiments^24,36,37^. New heads are regenerated within 20 days, with no significant changes in whole-body shape and size 6 weeks after amputation (Extended Data Fig. 2). This allowed us to consistently analyze tissue types from animals of different chronological ages and from animals that underwent amp–reg–feed cycles.

**Extended Data Fig. 3:**
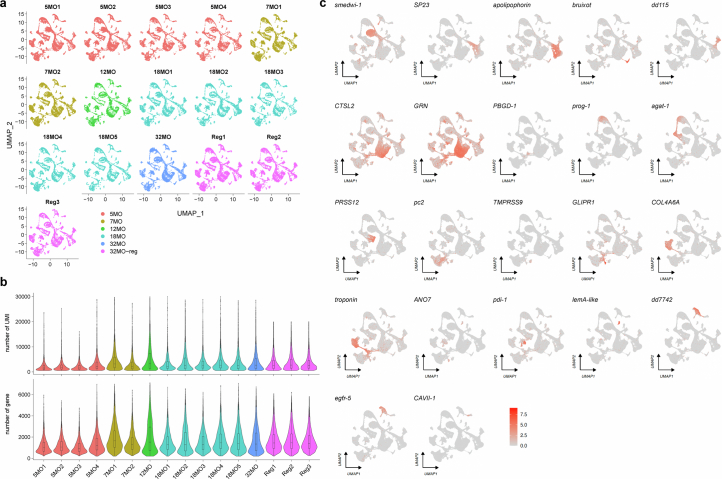
sc-RNA sequencing data quality control and cluster annotation. **(a**) UMAP plot depicting the distribution of cells from different replicates. Cells were colored by age. (**b**) Violin plots of the number of UMIs (top) and genes (bottom) of each sample. The captured cell number in each sample were indicated in Supplementary Table 1. The boxplots show the median (center), IQR (box) and whiskers extending to 1.5 × IQR. (**c)** Publicly reported tissue specific genes and their expression on the UMAP projection.

**Extended Data Fig. 4:**
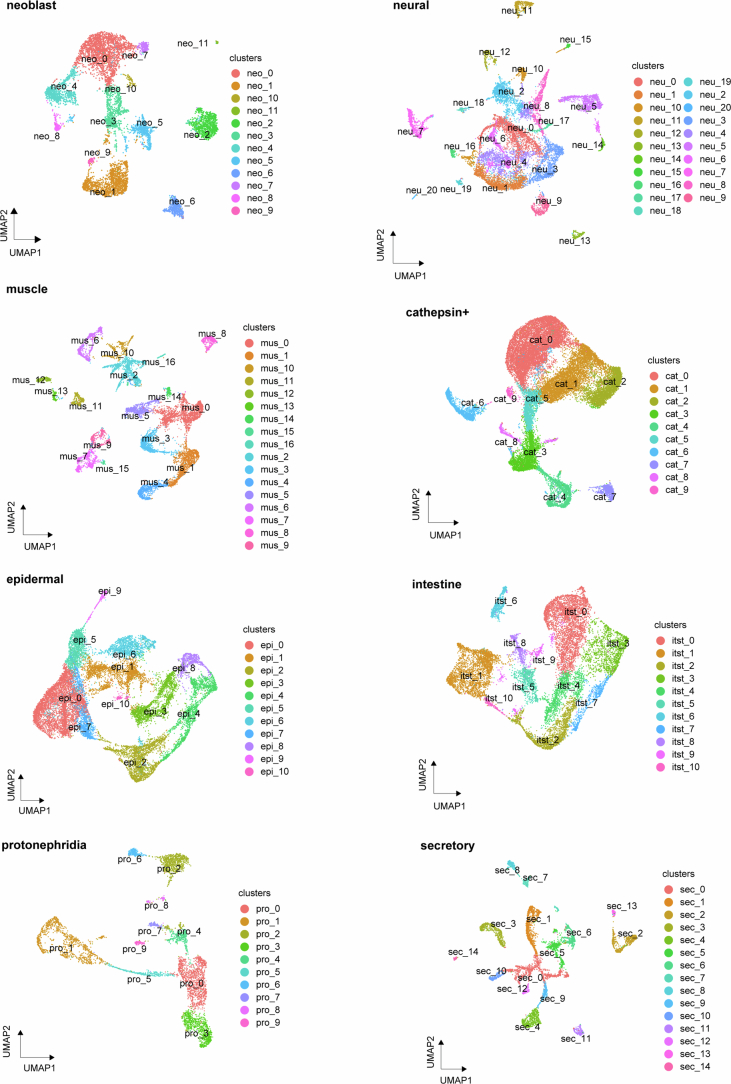
Subclustering of eight planarian tissues. UMAP plots of different tissues colored by sub-clusters. neoblast (12 clusters, 8,261 cells). neural (21 clusters, 13,187 cells). muscle (17 clusters, 10,361 cells). cathepsin+ parenchymal cell (10 clusters, 28,901 cells). epidermal (11 clusters, 18,532 cells). intestine (11 clusters, 14,050 cells). protonephridia (10 clusters, 5,123 cells). secretory (15 clusters, 6,202 cells).

We obtained a total of 104,617 high-quality single-cell transcriptomes from 16 heads at different ages and conditions: 5MO (*n* = 4), 7MO (*n* = 2), 12MO (*n* = 1), 18MO (*n* = 5), 32MO (*n* = 1) and 21MO–3Mpa–5Mpa–3Mpa (*n* = 3; Fig. 4a and Supplementary Table 1). We treated worms from multiple age groups and containers as young (5MO, 7MO) and old (12MO, 18MO, 32MO) to minimize environmental biases likely introduced during long-term housing. Unsupervised clustering of the data identified cells from all eight major tissue types^38,39^, including neoblasts (ASCs), epidermal cells, secretory cells, intestinal cells, protonephridia, cathepsin^+^ cells, neurons and muscle cells (Fig. 4b and Extended Data Figs. 3 and 4). With unsupervised subclustering of these eight tissue types, we identified and annotated 107 subclusters (Fig. 4c and Extended Data Fig. 4). Annotations of all subclusters are provided in Supplementary Table 2.

**Extended Data Fig. 5:**
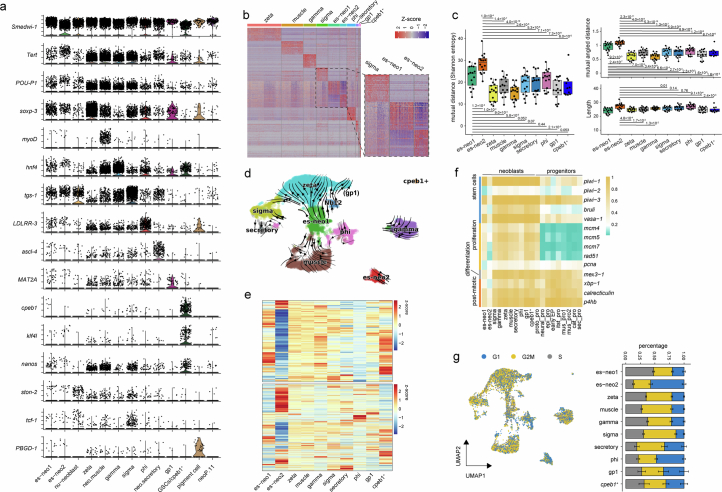
Identification and characterization of neoblast subclusters. (**a**) Publicly reported neoblast specific genes and their expression in different subclusters of the neoblast in the integrated dataset of current study and Issigonis et al.^56^. (**b**) Heatmap of top 30 genes expressed in neoblast subclusters, with unique patterns for es-neo cells. (**c**) Shannon entropy of different neoblasts. Each data point represents the mean value in one scRNA-seq sample from Fig. 4a, n = 16 samples were sampled. Two-sided Welch’s t-test. The boxplots show the median (center), IQR (box) and whiskers extending to 1.5 × IQR, observed minima and maxima (minima and maxima). (**d**) Velocities streamlines showing potential differentiation trajectories of neoblasts. (**e**) Heatmap showing the expression of 180 ‘unlimited primordial stem cells’ genes (top) and of whole transcriptome with a non-zero expression value (bottom) in neoblast subtypes, measured as the average of 16 samples. (**f**) Proportion of cells that express neoblast, proliferation, differentiation, and post-mitotic markers^91^. (**g**) UMAP projection of neoblasts with cells colored by predicted cell cycle state (left) and proportions of cells at each state. Each dot represents a single neoblast, n = 8261 neoblasts were examined. Error bars: standard error of the mean (SEM). Source data

We then characterized the stem cell populations, and found sexual adults harbored several stem cell subtypes that appeared homologous to stem cells in asexual animals, and hence likely have similar differentiation potentials. These include sigma (*soxP-1*^*+*^, *soxP-2*^*+*^), zeta (*soxP-3*^*+*^, *zfp-1*^*+*^, *egr-1*^*+*^, *p53*^*+*^), gamma (*hnf4*^*+*^, *gata456-1*^*+*^, *nkx2.2*^*+*^), nu (*tcf-1*^*+*^, *ston-2*^*+*^), muscle (*myoD*^*+*^), gp1, secretory and phi neoblast populations^38–42^ (Fig. 4c and Extended Data Fig. 5a). Gp1 neoblasts, as identified in ref. ^42^, highly express *MAT2A*, and likely comprise a group of gut progenitors distinct from the gamma neoblasts. Secretory neoblasts highly express *pdi-1* (ref. ^39^) and *ascl-4* (refs. ^38,42^). Phi neoblasts express high levels of *F-spondin*^43^, *LDLRR-3* (ref. ^43^) and *ETS-1* (ref. ^44^), which have been reported to regulate cathepsin^+^ parenchymal cells^38^. While 80.2% (±3.6%, s.e.m.) of sigma neoblasts are *tgs-1*^+^, 90.9% (±2.1%, s.e.m.) of *tgs-1*^+^ cells are *soxP-1*^+^ and *soxP-2*^+^ (sigma neoblasts). Tetraspanin-1, the antibody used to purify *tgs-1*^+^ ASCs in asexual planarians^42^, corresponds to 27.1% (±3.8%, s.e.m.) of sigma neoblasts (32.0% of *tspan-1*^+^ cells, ±2.2%, s.e.m.) and 14.3% (±1.3%, s.e.m.) of muscle progenitors (39.0% of *tspan-1*^+^ cells, ±2.3%, s.e.m.; Supplementary Table 3).

**Extended Data Fig. 6:**
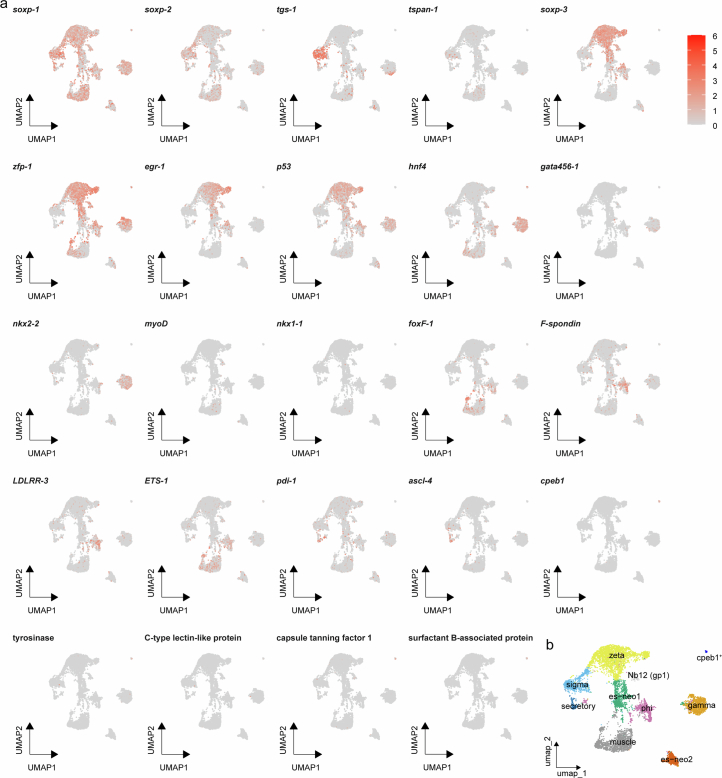
Expression of marker genes in neoblasts. (**a**) UMAP plots colored by expression of marker genes. (**b**) UMAP plot shows different neoblast subtypes.

We also identified previously uncharacterized stem cell clusters, which we named *cpeb1*^+^, es-neo1 and es-neo2 stem cells (Fig. 4c and Extended Data Figs. 5a and 6). Top genes expressed in *cpeb1*^+^ neoblasts (that is, tyrosinase, C-type lectin-like protein, capsule tanning factor 1 and surfactant B-associated protein) are all expressed in yolk glands^45^. Given that knockdown of *cpeb1* leads to the loss of yolk glands and ovaries^45^, *cpeb1*^*+*^ neoblasts likely represent progenitors of yolk glands. Due to the small number of *cpeb1*^*+*^ neoblasts (58 in all 16 samples; Supplementary Table 4), their aging processes were not examined in depth.

Computational analysis suggest es-neo stem cells are highly pluripotent. Es-neo stem cells show high expression of a range of canonical markers, including general markers for stem cells (for example, *smedwi-1*, *smedwi-2* and *smedwi-3*), and markers for tissue-specific stem cells (for example, sigma, zeta and gamma; Extended Data Fig. 5a). The expression of these marker genes is highly stochastic; the cells do not show consistent or deterministic marker expression like differentiated cells, and no markers are expressed in 100% of es-neo cells (Extended Data Fig. 5a,b). These gene expression patterns are reminiscent of the stochastic expression of pluripotency factors in embryonic stem cells (for example, *Nanog* and *Pou5f1* (*Oct4*); reviewed in ref. ^46^), a signature of pluripotency. We quantified the expression stochasticity with mean mutual distances, which is related to Shannon entropy and found that es-neo cells have higher or similar entropy among all subtypes of neoblasts (Extended Data Fig. 5c), consistent with high stochasticity. Some es-neo cells express *Smed-POU-P1*, a homolog of *Oct4* in planarians^47^, and the telomerase gene *Tert* at much higher levels (Extended Data Fig. 5a). *Oct4* encodes a transcription factor required for the formation of mouse embryonic stem cells^48^ and is one of the Yamanaka factors used to induce pluripotency^49^. *Tert* is required for telomere maintenance and long-term stem cell pluripotency and self-renewal^50^. Trajectory analysis with CellRank^51^ (mRNA splicing) supports the idea that es-neo1 may be a totipotent stem cell population that gives rise to zeta, sigma, phi, muscle, gamma, secretory, gp1 and *cpeb1*^+^ cells (Extended Data Fig. 5d). In addition, es-neo express at high levels, the deeply conserved 180-gene repertoire of unlimited primordial stem cells^52^ (Extended Data Fig. 5e). Es-neo1 and es-neo2 appear to have two complementary states, with genes most highly expressed in es-neo1 being more lowly expressed in es-neo2 and vice versa (Extended Data Fig. 5b). In contrary, other subtypes of stem cells express the top genes in es-neo with ~70% probability at intermediate levels. The complementary gene expression pattern between es-neo1 and es-neo2 is also true for the repertoire of 180 stem cell genes with conserved expression (Extended Data Fig. 5e) and the top 2,000 highly variable genes (HVGs) between all planarian cell types (Extended Data Fig. 5e). More es-neo1 cells express proliferation markers (for example, *mcm4*, *mcm5*, *mcm7* and *rad51*), similar to sigma, gamma, zeta and muscle neoblasts, than es-neo2 cells (Extended Data Fig. 5f). Cell cycle analysis showed es-neo2 had most cells in the G1 phase and the least number of cells in S phase (Extended Data Fig. 5g). In addition, es-neo2 have the least cells expressing the differentiation gene, *mex3-1* (ref. ^53^) and postmitotic genes^54^, compared to other neoblasts and tissue-specific progenitors (Extended Data Fig. 5f).

**Extended Data Fig. 7:**
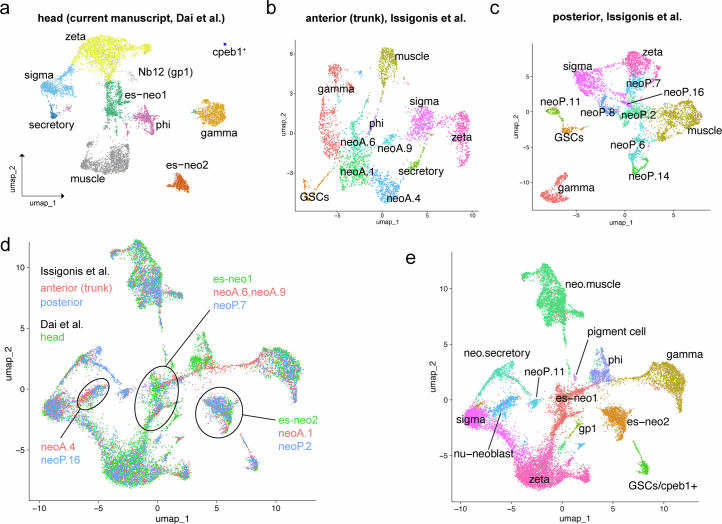
Neoblasts in the integrated analysis of Dai et al. and Issigonis et al. (**a**) Subclusters of neoblasts from Dai et al. Head tissues were used. (**b**) Subclusters of neoblasts from Issigonis et al. Trunk tissues were used and named as ‘anterior’. (**c**) Subclusters of neoblasts from Issigonis et al. Copulatory regions were used and named as ‘posterior’. (**d-e**) Subclusters of neoblasts after integration of the three datasets. Integration with labels of data and cell type sources (**d**). neoA.1 and neoP.2 are es-neo2 (**d, e**). neoA.6, neoA.9 and neoP.7 are es-neo1 (**d, e**). neoA.4 and neoP.16 are nu-neoblasts (**d, e**).

We were able to confirm the existence of es-neo1 and es-neo2 clusters in public single-cell datasets of asexual planarians^55^ (Supplementary Fig. 1), and trunks and tails of sexual planarians^56^ (Extended Data Fig. 7a–c). Unsupervised clustering of the neoblasts in anterior (trunks) and posterior (tails) of the dataset from Issigonis et. al.^56^ led to the identification of uncharacterized subclusters of neoblasts. After integration with our head dataset, we found these novel subclusters correspond to es-neo1 and es-neo2 stem cells (Extended Data Fig. 7d,e). The numbers of genes and unique molecular identifiers (UMIs) detected per es-neo cell were different from expectations of cell doublets, and were, instead, less than the numbers of genes and UMIs in every other previously reported subtype of neoblasts (Supplementary Fig. 2), suggesting es-neo are single cells, likely less active in transcription. Next, we extracted all *smedwi-1*^+^ cells from the integrated dataset and performed subclustering analysis. We were able to identify all known neoblast types (sigma, zeta, muscle, secretory, phi) and tissue-specific progenitors (intestine, cathepsin^+^, epidermal, neural). Es-neo cells did not form independent clusters and can be easily considered as part of sigma, zeta, epidermal or intestine cells (Supplementary Figs. 3 and 4). We were only able to locate es-neo cells with label transfer (es-neo1, es-neo2, neoA1, neoA6, neoA9, neoP2 and neoP7) from independent annotations, providing one potential explanation why es-neo cells were missing in earlier studies of planarian stem cells^38,39,42^.

**Extended Data Fig. 8:**
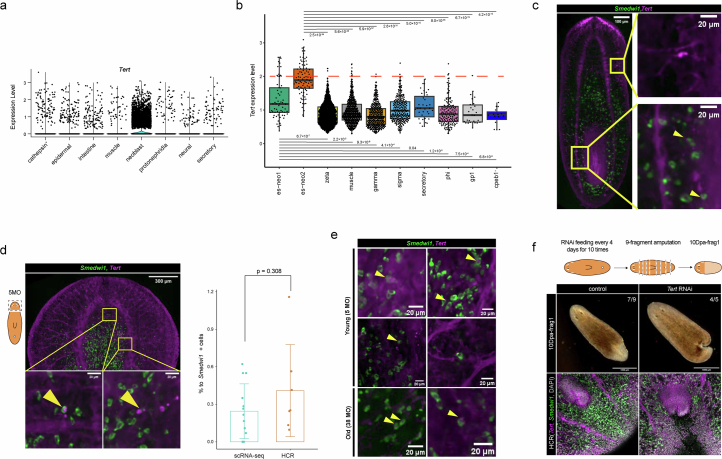
*Tert* expression in single cell analysis and HCR-FISH. (**a**) Violin plot showing the neoblast-specific expression of *Tert*. (**b**) Box plot showing *Tert* expression in various neoblast subclusters. Es-neo 1 and 2 have the most *Tert*^high^ cells (red dashed line: *Tert* expression level = 2). Each dot represents a *Tert*^+^ neoblast, n = 2488 neoblasts were examined. Two-sided Welch’s t-test. The boxplots show the median (center), IQR (box) and whiskers extending to 1.5 × IQR, observed minima and maxima (minima and maxima). (**c**) HCR-FISH detected a small subset of the *Smedwi-1*^+^ cells that expressed *Tert* at highest levels. Representative images of n = 3 individuals (**d**) HCR-FISH of *Smedwi-1* and *Tert* in the head of a 5MO worm. Bottom-left: *Smedwi-1*^+^ and *Tert*^high^ cells. Bottom-right: *Smedwi-1*^-^ and *Tert*^high^ cells. Representative images of n = 13 individuals. Percentage of *Tert*^high^ cells quantified from scRNA-seq and HCR-FISH data, normalized to number of *Smedwi-1*^+^ cells. Each dot represents a sample, *n* = 13, and 7 samples for scRNA-seq and HCR-FISH, respectively. Two-sided Welch’s t-test. Error bars represent mean ± SD. (**e**) Extra confocal images showing *Smedwi-1*^+^ and *Tert*^high^ cells in 5MO and 38MO planarians. Representative images of young (n = 3) and old (n = 4) individuals. (**f**) Head fragments at 10 days post amputation of control (n = 9) and *Tert* RNAi knockdown worms (n = 5). Top: regeneration defects in *Tert* RNAi. Bottom: *Smedwi-1* and *Tert* expression in posterior ends. Representative images of n = 4 individuals. Source data

To experimentally visualize es-neo stem cells, we performed fluorescence in situ hybridization (FISH) with the *Tert* probe because *Tert* expression is highly enriched in neoblasts (Extended Data Fig. 8a,b), and ~6–9% of the es-neo2 cells express *Tert* highly in all three datasets of head, anterior and posterior in scRNA-seq (Extended Data Fig. 8b and Supplementary Table 5). *Tert*^hi^ cells (*Tert* expression value > 2) only accounted for ~0.2–1% of other types of neoblasts in the head. In total, ~43.5–65.7% of *Tert*^hi^ cells were es-neo1 or es-neo2 cells in the scRNA-seq analysis (Extended Data Fig. 8b and Supplementary Table 5). In FISH experiments, we quantified the numbers of big bright cells that were likely *Tert*^hi^ cells in scRNA-seq (Extended Data Fig. 8c–e). The percentage of *Tert*^hi^ cells among *smedwi-1*^*+*^ cells in scRNA-seq (0.24% ± 0.06% s.e.m.) and the percentage of *Tert*^*+*^ cells among *smedwi-1*^*+*^ cells in FISH (0.41%, ±0.14% s.e.m.) were not significantly different (Extended Data Fig. 8d; Welch’s *t*-test). Consistently, this abundance is comparable to *smedwi-1*^+^ cells with long telomere length, quantified with telomere Q-FISH in whole sexual planarians^57^. Functionally, we found reduced expression of *Tert* by RNA interference led to reduced regeneration speed in head fragments (Extended Data Fig. 8f). While seven of nine head fragments formed tail blastemas 10Dpa in the control group, four of five head fragments with reduced *Tert* expression failed to form intact tail blastemas. As the blastema is formed by neoblast proliferation and differentiation^58^, this result suggests *Tert* plays a role in planarian stem cell function. Taken together, these data suggest es-neo are pluripotent stem cell populations in the planarians.

Es-neo1 showed enriched expression of ribosome genes (Supplementary Table 6). Es-neo2 showed higher mitochondrial content and lower mRNA counts (Supplementary Fig. 2 and Supplementary Table 6). We were only able to identify epi_3, a subcluster of epidermal cells, among all other tissues, which showed high mitochondria content as es-neo2 (Supplementary Fig. 5), suggesting this feature is very specific. As we cannot genetically label es-neo stem cells and track their differentiation, it remains challenging to definitively conclude if es-neo are real stem cell populations or analytic artifacts.

### Minimal changes detected in pluripotent ASCs

We next asked how aging affects stem cells and other cell types of the head. Most mammalian tissues, with the exception of the hematopoietic system^2^, exhibit an age-dependent decline in the total number of stem cells^2^. To quantify the proportion of stem cells in young and older planarians, we defined the ASCs as either the neoblast cluster, or *smedwi-1*^*+*^ cells. In our datasets, a slight decrease was observed, but we failed to detect a significant loss of stem cells with both definitions (Fig. 4d). In addition, we quantified *smedwi-1*^*+*^ cells with hybridization chain reaction fluorescence in situ hybridization (HCR-FISH) in 5MO and 38MO planarians, which did not show a significant decrease in *smedwi-1*^*+*^ cell density (Fig. 4e,f).

It was reported that aging in sexual planarians led to telomere shortening from 21.2 kb to 11.1 kb in 3 years and serial regeneration induced additional drastic shortening of telomeres^26^, which would imply loss of stem cells or replicative aging. We repeated this experiment with telomere restriction fragment analysis in sexual planarians of *S. mediterranea* that were 1MO, 5MO, 36MO and 5MO with three rounds of amp–reg–feed (Fig. 4g). In contrast to the previous report^26^, we did not observe measurable differences in telomere length as a result of aging, amputation or regeneration in sexual planarians. As all the telomere restriction fragments that we measured ran close to the resolution limit of the gel (~25 kb), we could not rule out minor changes in telomere length between the samples tested. We repeated this experiment in a colony of clones from the sexual lineage of *S. mediterranea* that were chronologically 20 years old, maintained by repeated amp–reg–feed^29^, with 6MO animals as controls, and in sexual planarians of *S. polychroa* that were 18MO, with 1MO animals as controls (Supplementary Fig. 6). Again, we failed to detect major changes in telomere length. Furthermore, our scRNA-seq dataset showed no significant changes in numbers (Fig. 4h), cell cycle (Fig. 4i) or entropy (Fig. 4j) among stem cell subtypes, except for a notable reduction in the G1 state of gamma cells in older ages. Taken together, these data are consistent with no significant loss of ASCs during planarian aging in 3 years.

**Fig. 5:**
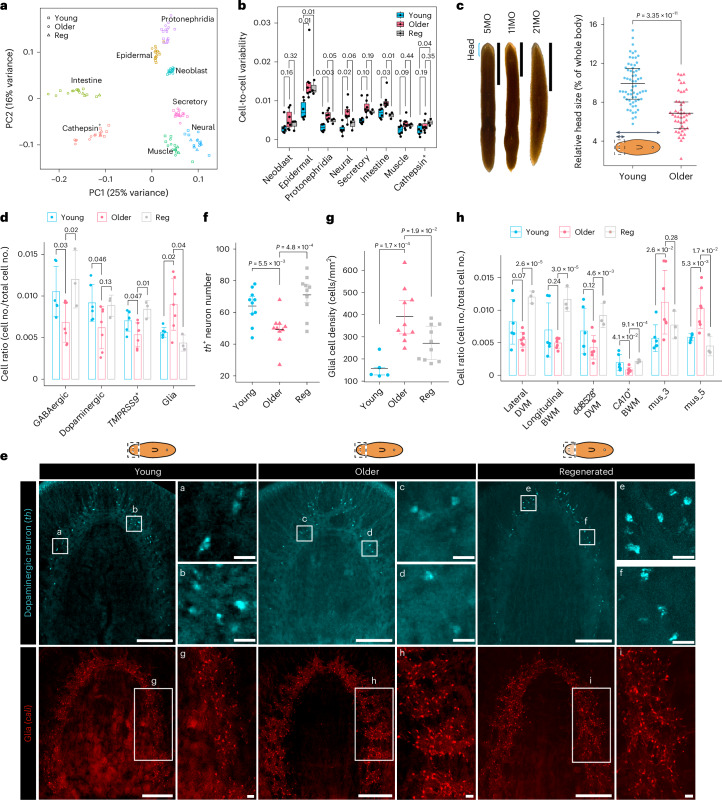
Tissue-specific aging and rejuvenation at the cellular level. **a**, PCA showing different degrees of dispersal by age and regeneration in stem cells and differentiated tissues. The union (4,321 genes) of the top 2,000 HVGs of each tissue was used for analysis. **b**, Cell-to-cell variability in various tissue types at different ages. Each data point represents the mean variability across all genes of a biological replicate derived from scRNA-seq data. The sample size for each experimental group is as indicated in Fig. 4a. Two-sided Welch’s *t*-test. The box plots show the median (center), IQR (box) and whiskers extending to 1.5 times the IQR. **c**, *S. mediterranea* exhibits progressive head atrophy with age (left). The blue bracket indicates the head region. Scale bars, 5 mm. The relative head size was defined as the fraction of the area of the head region to the whole worm area (right). Young group, <5MO (*n* = 63); older group, >12MO (*n* = 45). Two-sided Welch’s *t*-test. Error bar denotes the IQR; center line indicates the mean. **d**, Cell proportion analysis based on scRNA-seq data for neurons (GABAergic, dopaminergic, *TMPRSS9*^+^) and glia. The sample size for each experimental group is as indicated in Fig. 4a. One-way ANOVA with Tukey’s HSD test. Error bar denotes the mean ± s.d. **e**, HCR-FISH of dopaminergic neuron marker *th* (blue) and glia marker *cali* (red) in young (4–5MO), older (14–16MO) and old-regenerated (15MO–2Mpa) heads. Scale bars, 200 µm in head images and 20 µm in zoomed-in images. **f**, The number of *th*^+^ cells counted in the dorsal side of young, older and old-regenerated heads with HCR-FISH. Each data point represents an individual worm. *n* = 10 individuals for each experimental group. Two-sided Welch’s *t*-test. Error bar indicates the IQR; center line denotes the mean. **g**, Glia cell density in young, older and old-regenerated heads quantified from HCR-FISH images. Each data point represents an individual animal. *n* = 5, 10 and 10 individuals for young, older and regenerated groups, respectively. Two-sided Welch’s *t*-test. Error bar indicates the IQR; center line denotes the mean. **h**, Cell proportion changes of various muscle cell types in three age groups from scRNA-seq data. The sample size for each experimental group is indicated in Fig. 4a. One-way ANOVA with Tukey’s HSD test. Error bar indicates the mean ± s.d. *P* values or adjusted *P* values are indicated in the figures. DVM, dorsal ventral muscle. Source data

We found a dimension reduction method, principal component analysis (PCA), was sufficient to separate differentiated tissues of young samples and old samples, such as cathepsin^+^ parenchymal, protonephridia, epidermal and intestine (Fig. 5a). In these four tissue types, the regenerated heads of older worms clustered more closely with young heads, than with old heads (Fig. 5a).

While muscle, neural and secretory tissues were not separated into age groups by PCA, the neoblasts showed the least separation (Fig. 5a). In other words, the transcriptome of planarian ASCs showed the highest resistance to the impacts of aging or regeneration.

Cell-to-cell transcription variability increases during mouse aging^59,60^. The planarian scRNA-seq dataset allowed us to analyze cell-to-cell transcriptional variability during aging and rejuvenation. A significant increase in transcriptional variability in older planarians was detected in epidermal, protonephridia, neural and intestine cells, but not in ASCs. Interestingly, regeneration reversed transcriptional variability in intestinal cells, but not in epidermal and cathepsin cells (Fig. 5b). We conclude that cell-to-cell transcription variability increases with older age in differentiated tissues, but not significantly in ASCs. This variability can be reversed by regeneration in intestinal tissues specifically, with a strong trend in protonephridia and neural tissues.

### Regeneration reverses age-associated loss of neurons and muscle

Next, we set out to characterize age-associated changes in differentiated tissues (for example, neural, muscle). We observed a strong decrease in head size, as planarians get older. Relative head sizes decreased from 9.9% (±0.3%, s.e.m.) to 6.9% (±0.3%, s.e.m.) of the whole-body sizes (Fig. 5c). A significant decrease in GABAergic, dopaminergic and *TMPRSS9*^+^ neurons and an increase in glial cells in older planarians (12MO, 18MO and 32MO) were detected by scRNA-seq (Fig. 5d). Compared to young planarians (5MO, 7MO), older planarians lost 31.3% of the neurons in 3 of 20 neuronal cell types, resulting in a loss from 2.7% (±0.2%, s.e.m.) to 1.8% (±0.2%, s.e.m.) of the total cell numbers in the heads. Dopaminergic neurons can be identified by the expression of tyrosine hydroxylase (*th*)^61^. We validated the loss of *th*^*+*^ neurons and increase of *cali*^*+*^ glia^62,63^ in older planarian heads, and restoration of the cell composition in regenerated heads of older planarians with HCR-FISH (Fig. 5e–g). In the muscle, we detected significant loss of a subset of body wall muscle (BWM), which can be specifically identified by coexpression of *bwm1* and *CA10* genes (Fig. 5h). Unfortunately, *bwm1* and *CA10* transcripts localize in proximity with little overlapping, making quantification of this group of BWM cells challenging with HCR-FISH (Supplementary Fig. 7). In summary, the scRNA-seq dataset and experimental validation collectively detected statistically significant regeneration-induced reversal of age-associated changes in GABAergic, dopaminergic and *TMPRSS9*^+^ neurons, glial cells and *CA10*^+^ BWM muscle cells. Taken together, we conclude that planarian aging is accompanied by the alteration of tissue architecture, which can be mitigated by regeneration.

### Regeneration reverses age-dependent molecular changes

**Fig. 6:**
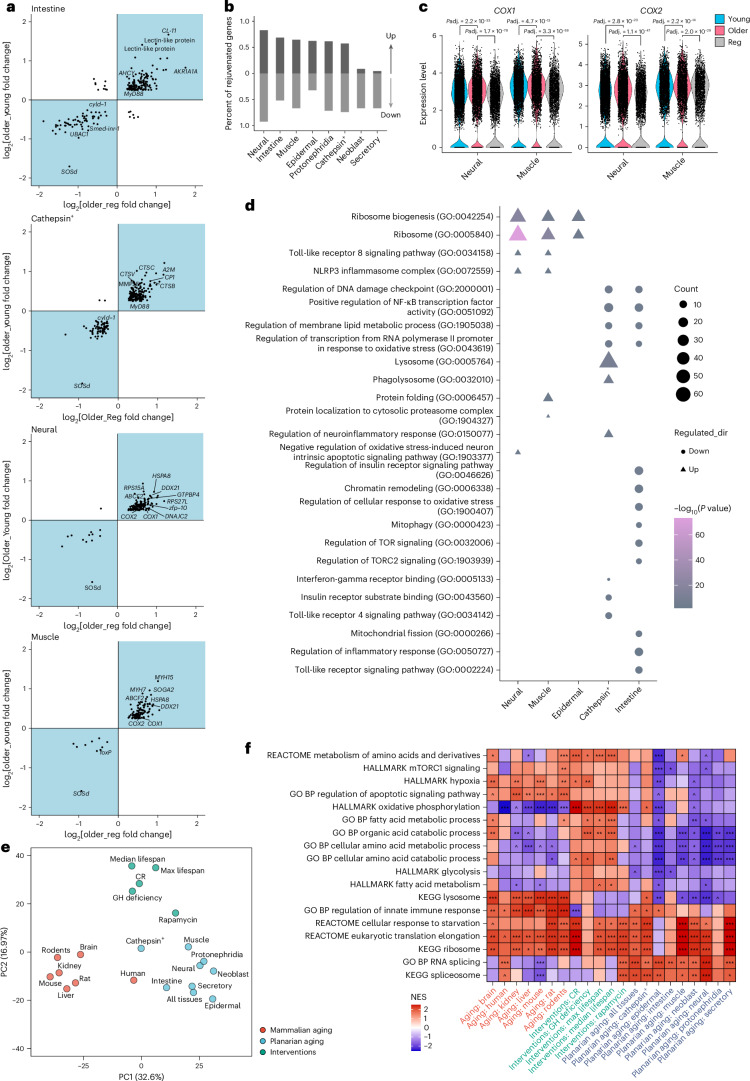
Reversal of aged-dependent molecular changes. **a**, log_2_-transformed fold changes of PAGs during aging (*y* axis, older versus young) and after regeneration (*x* axis, older versus regenerated) in different tissues based on scRNA-seq data. Top-right quadrant represents genes upregulated in older conditions, compared to either young or regenerated conditions. **b**, Percentage of rejuvenated genes in each tissue. Dark-gray bars represent reversed PAGs that were upregulated in older age; light-gray bars represent PAGs that were downregulated in older age. **c**, Expression level changes of *COX1*/*COX2* in neural and muscle tissues based on scRNA-seq data. The sample size for each experimental group is indicated in Fig. 4a. Two-sided Wilcoxon rank-sum test *P* value adjusted using Bonferroni correction. **d**, Enriched Gene Ontology (GO) terms among rejuvenated genes in various tissues. Two-sided chi-squared test (when all expected frequencies are greater than 5) or Fisher’s exact test *P* value adjusted with Benjamini–Hochberg correction. **e**, PCA of gene expression signatures of planarian aging (blue), mammalian aging (red) and lifespan-extending interventions in mice (green). Variance explained by first two principal components (PC1 and PC2) is indicated in parentheses. CR, caloric restriction; GH, growth hormone. **f**, Statistically significant pathways associated with planarian aging (blue), mammalian aging (red) and lifespan-extending interventions in mice (green). GSEA permutation test *P* value adjusted for multiple comparisons with Benjamini–Hochberg correction. Only functions significantly enriched by at least two signatures are visualized (adjusted *P* value < 0.1). Cells are colored based on normalized enrichment scores (NES). ^*P* adjusted < 0.1; *P adjusted < 0.05; **P adjusted < 0.01; ***P adjusted < 0.001. BP, biological process. Source data

**Extended Data Fig. 9:**
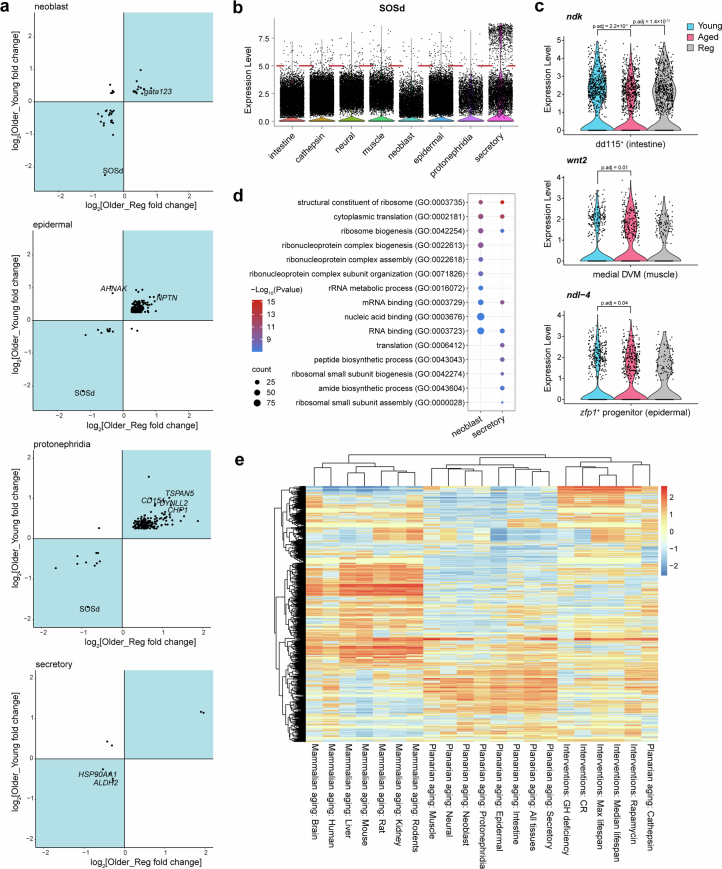
Age-associated molecular changes. (**a**) Log_2_-transformed fold changes of PAGs during aging (y-axis, older v.s. young) and after regeneration (x-axis, older v.s. regenerated) in different tissues (neoblast, epidermal, protonephridia, and secretory) based on scRNA-seq data. Top right quadrant represents genes upregulated in older conditions, compared to either young or regenerated conditions. (**b**) Expression level of gene SOSd in different tissue types. (**c**) Expression of positional control gene *ndk*, *wnt2*, and *ndl-4* during aging and after regeneration. Two-sided Wilcoxon Rank Sum test, p-value adjusted using Bonferroni correction. (**d**) Top 10 enriched terms from Gene Ontology enrichment analysis of non-rejuvenated up-regulated PAGs in neoblast and secretory cells. Two-sided Chi-square test (when all expected frequencies are greater than 5) or Fisher’s exact test, p-value adjusted with Benjamini-Hochberg correction (**e**) Heatmap of normalized enrichment scores (NES) obtained from functional enrichment analysis (GSEA) of gene expression signatures of planarian aging, mammalian aging and lifespan-extending interventions in mice. Cells are colored based on NES. Hierarchical clustering with Euclidean distance metric and complete linkage was utilized to cluster signatures and functions.

Next, we sought to identify age-associated transcriptional changes and determine if regeneration reverses such changes across tissues. To do so, we examined changes in gene expression between young, old and regenerated planarian heads. For convenience, we will name planarian genes with significant age-associated changes in expression as planarian age-associated genes (PAGs, |log_2_fold change (FC) | > 0.25, adjusted *P* value < 0.05). PAGs were observed in all tissues, but the degree and pattern of gene expression changes showed tissue specificity (Fig. 6a,b, Extended Data Fig. 9a and Supplementary Table 7). Consistently, ASCs had fewer PAGs relative to differentiated tissues (Fig. 6a,b and Extended Data Fig. 9a). Regeneration reversed the expression of a significant portion of the PAGs in all tissues examined, from 4.5% to 82.9% for upregulated PAGs in older age, and from 32.3% to 92.3% for downregulated PAGs in older age (Fig. 6a,b and Extended Data Fig. 9a).

The PAGs observed were either widespread across various tissues or specific to individual tissues (Fig. 6a–d). For example, we identified and named a gene as *SOSd*, which was downregulated in most tissues in older planarians (Fig. 6a and Extended Data Fig. 9a,b). The highly conserved mitochondrial genes cytochrome c oxidase subunit 1 and 2 (*COX1/COX2*) were specifically upregulated in neural and muscle tissues (Fig. 6a,c) and play important roles in oxidative phosphorylation and neuronal and muscle health in older age^64,65^. Changes in the expression of position control genes (for example, *ndk*, *wnt2* and *ndl-4*) were observed in older planarians (Extended Data Fig. 9c), which may impact aging of the planarian eyes. Age-associated downregulation of *SOSd* and *ndk* and upregulation of *COX1/COX2* were all reversed by regeneration (Fig. 6a,c and Extended Data Fig. 9a).

The specific molecular changes revealed by our gene expression analysis indicate parallels in the effects of aging between sexual planarians and mammals^66^, and here, too, there are tissue-specific effects^67^ (Fig. 6d, Extended Data Fig. 9d,e and Supplementary Table 8). For example, computational analysis suggested upregulation of planarian genes in neural and muscle tissues that are related to mammalian genes in inflammation and the innate immune response, and suggested downregulation of insulin/target of rapamycin complex-1/complex-2 signaling and DNA damage response-related genes in cathepsin^+^ parenchymal and intestinal cells. Other notable biological processes altered in planarian aging include proteostasis (lysosome, protein folding), transcription and translation, oxidative stress, chromatin remodeling and mitochondria regulation, all of which are hallmarks of aging^1,35^. Specifically, our dataset suggests expression of the insulin receptor *Smed-inr-1* (ref. ^68^) was downregulated in the intestine and rejuvenated after regeneration (Fig. 6a,d). Expression of proteostasis-related genes (for example, *Hspa8*) was upregulated in neural and muscle tissues and was rejuvenated after regeneration (Fig. 6a,d). These results highlight conserved aspects of the aging process and underline tissue-specific patterns.

### Comparative analysis with mouse and human aging

To directly compare planarian aging to mammalian aging (that is, mice, rats and humans), we treated the single-cell sequencing data as pseudo-bulk and used gene-set enrichment analysis (GSEA) to compare with datasets of mouse, rat and human aging and datasets of mouse lifespan-extending interventions^69–71^ (Supplementary Table 9). In general, signatures of aging from planarians were distinct from mammalian aging and were closer to signatures of lifespan-extending interventions at the level of enriched functions (Fig. 6e and Extended Data Fig. 9e). Remarkably, the planarian aging signature was closer to the human signature than to the aging signatures of mice and rats (Fig. 6e).

Functional enrichment analysis revealed some pro-aging mammalian mechanisms in aged planarian tissues (Fig. 6f), such as downregulation of genes involved in fatty acid metabolism and oxidative phosphorylation (especially in epidermal and neoblast cells) and upregulation of genes involved in the innate immune response, translation elongation and ribosome organization. Functional enrichment analysis also suggested that older planarians exhibit some antiaging mammalian features, such as upregulation of genes related to mRNA splicing and downregulation of genes associated with hypoxia, apoptotic response, mTORC1 signaling and lysosome function (Fig. 6f). Downregulation of mTORC1 signaling leads to increased telomere length^57^. This is consistent with our finding that aging in sexual planarians is not accompanied by telomere shortening (Fig. 4g). Taken together, our analysis suggests aging in planarians is associated with pro-aging and antiaging mechanisms, with shared and distinct mechanisms when compared to mice and humans.

### Head regeneration reverses the aging transcriptome in distal tail tissues

**Fig. 7:**
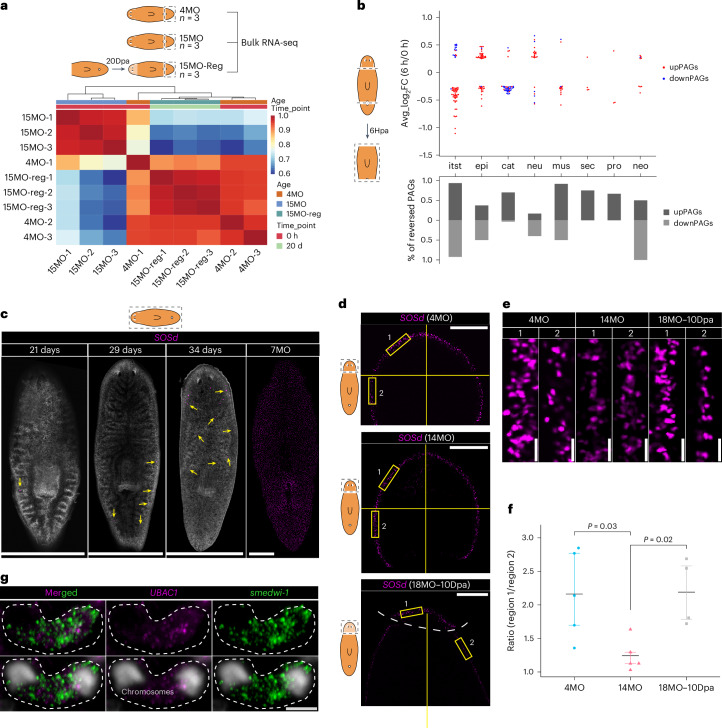
Potential rejuvenation mechanisms. **a**, Heat map of Pearson correlation coefficients of tail transcriptomes between young (4MO), old (15MO) and head-regenerated (15MO–20Dpa) conditions showing rejuvenation in distal uninjured tissues. Three biological replicates were used for each condition. **b**, log_2_-fold change of upPAGs (red) and downPAGs (blue) during the early stage of regeneration (6Hpa versus 0Hpa) in different tissues. Percentage of reversed PAGs at 6Hpa. Hpa, hours post amputation; itst, intestine; epi, epidermal; cat, cathepsin^+^; neu, neural; mus, muscle; sec, secretory; pro, protonephridia; neo, neoblast. **c**, Confocal images showing *SOSd*^+^ cell density at different ages. Representative images of *n* = 3 animals for each age. Yellow arrows point to the *SOSd*^high^ cells. Scale bars, 1 mm. **d**, Distribution of *SOSd*^+^ cells in young (4MO), old (14MO) and regenerated (18MO–10Dpa) heads. Representative images of *n* = 5 (4MO), *n* = 5 (14MO) and *n* = 4 (18MO–10Dpa) animals. Yellow rectangles indicate selected regions for cell density quantification. Vertical yellow line indicates the midline of the animal; horizontal yellow line indicates the widest points from the left to right sides of the head; white dashed line indicates the boundary between blastema and old trunk tissues, marked by two notches introduced after animal fixation. Scale bars, 500 µm. **e**, Representative images of regions 1 and 2 from 4MO, 14MO and 18MO–10Dpa heads. Region size, 273.45 μm × 79.55 μm. Scale bars, 20 μm. **f**, Quantification of cell density ratio between region 1 and region 2. Each data point represents an individual worm. *n* = 5, 5 and 4 animals for 4MO, 14MO and 18MO–10Dpa, respectively. Two-sided Welch’s *t*-test. Error bar indicates the IQR; center line denotes the mean. **g**, Confocal images showing expression of *UBAC1* and *smedwi-1* in a pair of dividing stem cells. Representative images of *n* = 3 animals. Dividing stem cells are marked by two blobs of condensed chromosomes (gray color). Scale bar, 10 μm. Source data

**Extended Data Fig. 10:**
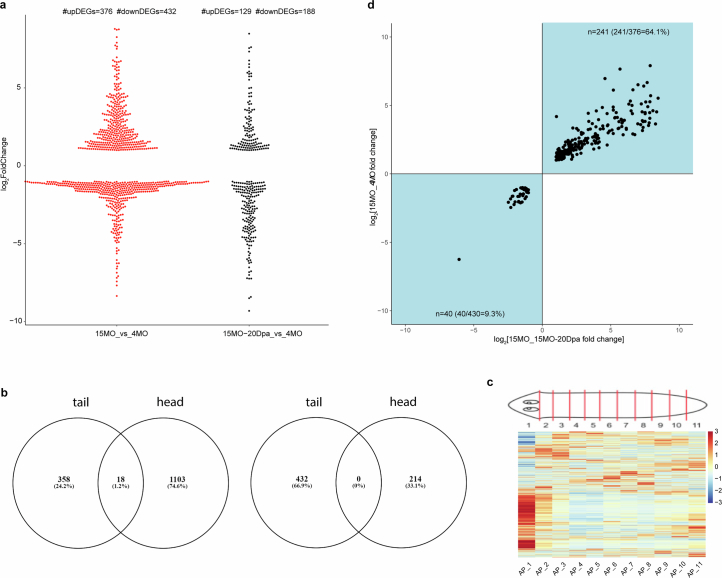
Rejuvenation of transcriptomic profiles in distal tails. (**a**) Differentially expressed genes in old and old-regenerated tails compared with young tails, two-sided Wald test, p-value adjusted with Benjamini-Hochberg correction, adjusted p-value < 0.05, |log_2_FC| > 1. (**b**) Overlap of PAGs in tail and head (left: up-regulated, right: down-regulated). (**c**) Heatmap of expression profiles along planarian A/P axis showing distinct expression patterns of head and tail^59^. (**d**) Log_2_-transformed fold changes of DEGs during tail aging (y-axis, 15MO v.s. 4MO) and after head regeneration (x-axis, 15MO v.s. 15MO-regenerated). The expression patterns of genes that distributed in blue region were reversed after head regeneration. Source data

Our experiments on the effects of aging and regeneration of the eyes (Figs. 1 and 2), fertility and motility (Fig. 3) and whole-animal ROS (Fig. 3) collectively suggest global tissue rejuvenation after regeneration. To provide direct evidence, we examined if head regeneration results in rejuvenation of tails that are distant from the injury site (Fig. 7a). We removed heads from 15MO planarians and allowed the rest of the body to regenerate the missing heads for 20 days (15MO–20Dpa). We then collected tails from young (4MO), older (15MO) and 15MO–20Dpa planarians and sequenced their transcriptomes. Correlation analysis showed that tails from 15MO–20Dpa planarians had higher similarity to the 4MO group than to the 15MO group (Fig. 7a). Expression of 64.1% and 9.3% of upregulated and downregulated genes, respectively, in 15MO tails was reversed after head regeneration, even though the tail transcriptome is drastically different from the head transcriptome^72^ and few PAGs were shared (Extended Data Fig. 10). Hence, we propose that rejuvenation happened in distal uninjured tissues, and regeneration leads to global rejuvenation.

To determine how regeneration led to rejuvenation of PAG expression in older planarians, we compared PAGs to a list of genes that were altered by regeneration in sexual planarians in a public dataset^73^ (Fig. 7b and Supplementary Table 10). Remarkably, in intestinal, epidermal, neural, muscle and neoblast tissues, 16.7% to 93.5% of the PAGs, shared by the 6-h injury response, reversed their expression to the opposite direction. A significant portion of the upregulated PAGs, 66.7% to 75.0%, but not the downregulated PAGs, reversed their expression in cathepsin^+^, protonephridia and secretory cells. This suggests that regeneration-induced rejuvenation has temporally distinct mechanisms among different tissues.

### Novel biology implicated by the study of PAGs

Tissue rejuvenation involves restoration of age-altered cell distribution patterns. One PAG that is downregulated in multiple tissues, *SOSd* (Fig. 6a), showed an intriguing expression pattern (Fig. 7c). Before sexual maturation, *SOSd*^hi^ cells increased from ~1 cell in 21-day-old hatchlings, to ~5 cells (±1 cell, s.e.m.) in 29-day-old hatchlings, and to ~151 cells (±114 cells, s.e.m.) in 34-day-old hatchlings (Fig. 7c). After reaching sexual maturity, *SOSd*^hi^ cells occupied the entire dorsal and ventral domain (Fig. 7c and Supplementary Video 1). At the dorsoventral tissue boundaries, the density of *SOSd*^hi^ cells was higher in the head than in the trunk in 4MO planarians. This ratio decreased in 14MO planarians. During regeneration, the density of *SOSd*^hi^ cells in the regenerating head blastema was not restored by 5Dpa, but was restored by 10Dpa, and resulted in a head–trunk distribution pattern recapitulating 4MO planaria (Fig. 7d–f and Supplementary Fig. 8). The timing differences between restoration of gene expression (Fig. 7b) and restoration of *SOSd* pattern (Fig. 7d–f) suggest different aspects of the planarian physiology can be rejuvenated at different time points of regeneration.

Another PAG, a homolog of human *UBAC1* that is a subunit of an E3 ubiquitin–protein ligase complex, was downregulated in intestinal cells (Fig. 6a). We found that 6 h after injury, even before the formation of the blastema, *UBAC1* RNA was asymmetrically distributed into one of the two daughter cells of dividing *smedwi-1*^+^ stem cells in the trunk (Fig. 7g). The cell that inherited more *UBAC1* retained more of the stem cell marker *smedwi-1*.

## Discussion

Our studies revealed a naturally evolved solution to reverse age-associated physiological decline in planarians. We found, within 3 years after birth, sexual planarians developed age-associated physiological, cellular and molecular changes that are consistent with our current understanding of aging. Interestingly, pluripotent ASCs do not show substantial age-related changes compared to the differentiated tissues evaluated by transcriptome states and numbers, which may be able to contribute to extreme longevity through cell turnover^74^. We also found that regeneration reverses the effects of aging on multiple tissues, hundreds of genes and several physiological traits. In addition, regeneration-induced rejuvenation is not restricted to newly regenerated tissues but can be global and is found in tissues distant from the wound site.

To facilitate future studies of rejuvenation mechanisms, we propose to use nt-rejuvenation (new tissue rejuvenation) to describe experimental designs examining newly regenerated tissues (for example, Fig. 4a), and to use ot-rejuvenation (old tissue rejuvenation) to describe experimental designs examining tissues that do not require regeneration (for example, Fig. 7a). Experiments in Figs. 1e, 2 and 3 involve both nt-rejuvenation and ot-rejuvenation. As our data support that older planarians are capable of nt-/ot-rejuvenation in multiple tissues and hundreds of genes, leading to rejuvenation of whole-body physiological states (for example, fertility, oxidative stress), we propose to describe this phenomenon as global rejuvenation, in comparison to rejuvenation studies in a few specific cell types or one specific tissue.

The biology of aging in long-lived animals is understudied^22^. Our work here in planarians demonstrates that the extreme longevity of planarians is not due to an exceptionally slow aging rate, at least not in the first 3 years of their lifespan. With the longest-living laboratory colony being 20 years old, it is expected that pro-longevity mechanisms are activated in older planarians, as is supported by our comparative analysis with mouse, human and lifespan extension interventions. Identification of such pro-longevity mechanisms, and determining how they involve stem cells, would be helpful to understand the basic biology of aging, and comparative analysis with mammals^75–79^. For example, both our dataset and mouse datasets^77,80^ suggest prominent roles of mitochondria in aging and highlight its responsiveness in rejuvenation.

Our data showed minimal changes in pluripotent ASC (neoblast) numbers and a more stable transcriptome state in the ASCs compared to differentiated tissues in older planarians. However, the differentiation capacity of ASCs is likely altered in planarian aging as we observed an increase and a decrease in specific cell types in multiple differentiated tissues. We cannot be certain of this possibility without showing that differentiated cell types die at similar rates during aging. Likewise, the committed tissue progenitors may show stronger aging phenotypes than the more pluripotent ASC compartment. Nonetheless, the extreme longevity suggests changes in differentiated cell types are nonlethal but possibly can lead to spontaneous degeneration and injury-like responses to induce rejuvenation.

Collectively, our work establishes planarians as an easily accessible and genetically tractable long-lived animal model for studies of aging and rejuvenation. Our work is limited by the number of aging assays that we can perform in one study. Future investigations of genomic instability, mitochondrial function, telomere regulation and stem cell functions would provide more clarity on the aging and antiaging processes achieving extreme longevity and rejuvenation in planarians. Whether such antiaging processes function as a mechanism to achieve agelessness in fissioning planarians will also be an interesting question^81^.

## Methods

### Ethics

Our work complies with all relevant ethical regulations under IBCA00002193, approved by the Institutional Biosafety Committee of the University of Michigan.

### Planarian strains, husbandry and fertility

All eggs and animals of *S. mediterranea* were maintained in 1× Montjuich salts, in plastic containers, at 18 °C as previously described^82^. Planarians were fed with organic cow liver weekly. Egg capsules were collected weekly. Collection dates of capsules were used as birth dates of the hatchlings. The inbred strains examined include A5G, D5D, D2E, S2F8b, S2Fn and LAF. Animals in the current study were matched for size (Supplementary Table 11) and starved for a week. The wild planarians of *S. polychroa* were a gift from M. R. León after a field trip to Sardinia. The species was identified by its head, eye morphology, genome and karyotyping^83^.

For static culture, a medium plastic Tupperware container (4.25 inches wide × 4.25 inches long × 3.06 inches high, 473 ml (16 oz), Ziploc) houses ~5–10 animals, and a large Tupperware container (5.87 inches wide × 9 inches long × 3.25 inches high; 2,130 ml (72 oz), Ziploc) houses ~20–50 animals. Tupperware containers were filled with sterile planarian water at least two-thirds full. Water was changed once a week after feeding.

Fertility was calculated as the percentage of egg capsules that hatched divided by the total number of egg capsules collected over a defined period. The number of hatchlings and the number of hatched egg capsules were documented for each batch of egg capsules 3 weeks after collection. To reliably compare fertility across strains or ages, fertility data from the same amount of time were binned as one data point. Multiple containers of such groups of animals were used as replicates to account for variability.

### Nomenclature

For clarity, repeatability and interpretability, we propose to use consistent nomenclature for regenerated planarians in experiments. For the name 21MO–3Mpa–5Mpa–3Mpa, planarians went through three rounds of amputation and regeneration. The first round of amputation happened when the worms were 21MO. Three months after the first amputation, they went through a second round of amputation. Five months after the second amputation, they went through a third round of amputation. Three months after the third amputation, they were used for experiments. Chronologically, the animals were 32MO. Methods of amputation (for example, head removal, three fragments or nine fragments) were supplied as cartoons alongside the figures presenting the data. Animals were fed once a week. Feeding was resumed by 20Dpa.

### scRNA-seq library preparation

Planarian heads were surgically removed along the posterior end of the auricle. Tissues were macerated with a surgical knife in CMF buffer supplemented with 0.5% BSA (CMFB) as previously described^42^. Macerated tissues were kept on ice-cold CMFB in a 6-cm Petri dish with gentle horizontal shaking for 20 min. Tissues were pipetted up and down every 5 min with a transfer pipette five to seven times to ensure sufficient disassociation and optimal viability. Cells were filtered through a 40-μm strainer before collection by spinning at 400 rcf for 5 min at 4 °C. Cells were counted manually. Cell viability was assessed by Trypan blue (0.4%). A viability reading of more than 70% is required before library preparation. The single cells were captured on the Chromium Controller (10x Genomics) and libraries were prepared using the Chromium Single Cell 3′ Gel Bead and Library Kit (10x Genomics). Libraries were sequenced on NovaSeq S4 platform (Illumina) with 150-bp paired-end reads.

### scRNA-seq data analysis

Sequencing data were preprocessed and mapped to the reference genome^30,84^ using Cell Ranger software v6.0.1 (10x Genomics). The count pipeline was used to generate the gene expression matrix. Cells with less than 500 detected genes, and high expression levels of mitochondrial genes were removed (Supplementary Table 1). Doublets were removed from each sample by identifying outliers with extremely high UMIs and by using DoubletFinder^85^. In total, 104,617 cells were kept for subsequent analysis. The UMI counts of each cell were normalized by Seurat^86^, and log transformed for further analyses and visualization. The top 2,000 genes with the highest standard deviations were identified as HVGs and were used for dimension reduction. Significant principal components were identified with the elbow method, followed by a graph-based approach (Shared Nearest Neighbor) to cluster cells. UMAP was used to visualize the clusters in two-dimensional space.

To integrate different samples, the Seurat anchor-based method^87^ was used to correct batch effects between datasets. The package Clustree^88^ and known biological markers were used to determine the optimal resolution for clustering, resulting in 51 distinct clusters (tissue level). The cluster markers were calculated using the FindAllMarkers function in Seurat, and clusters expressing known tissue-specific markers were assigned to the same tissue type. In addition, clusters were compared to the planarian cell atlas^38,39^. Clusters with high similarity with known tissue lineages were then considered as the same tissue types.

Cells of each tissue were subset from the whole dataset and integrated following similar processes to those described above. Most subclusters were assigned to previously described cell types according to shared expression of known markers. For those without known markers, the top specifically enriched genes were used to label the subclusters (Supplementary Table 2).

Analyses of published scRNA-seq datasets of sexual and asexual planarians^55,56,73^ followed a similar approach.

### Trajectory inference analysis

Velocyto^89^ (version 0.17.17) was used to quantify spliced and unspliced mRNA. scVelo^90^ (version 0.2.5) was used to compute RNA velocity vectors based on splicing ratios using the steady-state model on a per-cell basis. CellRank^51^ (version 1.5.1), combining the robustness of trajectory inference with directional information from RNA velocity, was used to compute the initial and terminal states.

### Mean mutual distances and Shannon entropy

A quantity related to Shannon entropy, which we call mean mutual distances, was used to measure stochastic gene expression of neoblasts and as a parameter for estimating the stemness of various neoblast subtypes. The normalized expression values of the top 2,000 HVGs of all neoblasts were used for calculation.

For each cell, we associate a vector:

$${\bf{v}}=\left({n}_{1},\ldots ,{n}_{2,000}\right)$$

to that cell, where ${n}_{1},\ldots ,{n}_{2,000}$ are the normalized gene expression values of the ${1}^{\mathrm{st}}$,…, ${2,000}^{\mathrm{th}}$ gene.

For a cell with vector ${\bf{v}}=({n}_{1},\ldots ,{n}_{2,000})$, its length is defined to be:

$$\left|{\bf{v}}\right|\equiv \sqrt{{n}_{1}^{2}+\ldots +{n}_{2,000}^{2}}$$

For two cells with vector ${\bf{u}}=({\bf{m}}_{1},\ldots ,{\bf{m}}_{2,000})$ and ${\bf{v}}=({\bf{n}}_{1},\ldots ,{\bf{n}}_{2,000})$, their mutual distance is defined to be:

$$d({\bf{u}},{\bf{v}})\equiv \left|{\bf{u}}-{\bf{v}}\right|=\sqrt{{\left({\bf{m}}_{1}-{\bf{n}}_{1}\right)}^{2}+\ldots +{\left({\bf{m}}_{2,000}-{\bf{n}}_{2,000}\right)}^{2}}$$

Their mutual angle is defined to be:

$$\theta\left({\bf{u}},{\bf{v}}\right)\equiv {\rm{ArcCos}}\frac{{m}_{1}{n}_{1}+\ldots +{m}_{2,000}{n}_{2,000}}{\sqrt{{n}_{1}^{2}+\ldots +{n}_{2,000}^{2}}+\sqrt{{m}_{1}^{2}+\ldots +{m}_{2,000}^{2}}}$$

The plots are generated in the following way. Collect all vectors ${\bf{v}}_{1},\ldots ,{\bf{v}}_{s}$ within a given pair of cell type and sample. The length data is the set:

$${\rm{Length}}=\left[\left|{\bf{v}}_{i}\right|,1\le i\le s\right]$$

The mutual distance data is the set:

$${\rm{Mutual}}\,{\rm{distance}}=\left[d\left({\bf{v}}_{i},{\bf{v}}_{j}\right),1\le i < j\le s\,\right]$$

The mutual angle data is the set:

$${\rm{Mutual}}\; {\rm{angle}}=\left[\theta \left({\bf{v}}_{i},{\bf{v}}_{j}\right),1\le i < j\le s\,\right]$$

The length of the vector quantifies the transcription activity of the cells. Longer length implies the cells express more genes at higher levels. The mutual angle between two vectors quantifies the similarity of the two cells. More stochasticity in gene expression of the examined cells leads to larger mutual angles. The mutual distance between two vectors is a combined measure of cell activity and gene expression stochasticity. We calculated the mean of the mutual distances between all pairs of cells for each cell type of each sample. To see their relation, consider a *D* dimensional Gaussian distribution:

$${x} \sim {\mathscr{N}}\left(\,{{\mu }},\sum\right),$$

where ${{\mu }}$ is the mean and ${{\sum }}$ is the covariance matrix. The Shannon entropy for this distribution is

$$H=\frac{D}{2}\left(1+\mathrm{ln}2\pi \right)+\frac{1}{2}\mathrm{ln}\det \left({{\sum }}\right).$$

The mean mutual distance is

$$E\left(\left|x-y\right|\right)\cong \sqrt{E\left({\left|x-y\right|}^{2}\right)}=\sqrt{2{\rm{Tr}}\left(\sum \right)}.$$

Both the Shannon entropy and the mean mutual distance are functions that increase with larger covariance ${{\sum }}$, characterizing more stochasticity in gene expression.

### Cell cycle analysis

The cell cycle state (G1, G2/M, S) of neoblasts was computationally predicted by the expression of cell cycle-associated markers^91^ (Supplementary Table 12). The CellCycleScoring function in Seurat was performed with default parameters to assign the S and G2M cell cycle score to each cell, along with the phase assignment in the G1, G2M or S phases based on the cell cycle score.

### Differential gene expression analysis and GO enrichment analysis

For cell clusters, differentially expressed genes (DEGs) between young versus aged, and aged versus regenerated, were identified using the FindMarkers function in Seurat^86^, which uses the Wilcoxon rank-sum test with *P* values adjusted by Bonferroni correction. Significant DEGs were identified with thresholds |log_2_FC | > 0.25 and an adjusted *P* value < 0.05. The DEGs of each tissue between 0 h and 6 h samples from the published scRNA-seq datasets (CRX495534, CRX495535) were identified as described above.

GO enrichment analysis was conducted as follows: (1) Functional annotation of all genes was performed by BLAST against the Swiss-Prot database; (2) GO term assignment per gene was obtained by extracting the GO IDs of the best BLAST hit; and (3) EnrichPipeline^92^ was used to identify enriched GO terms among a given gene set. The algorithm uses a ‘chi-Fisher’ test, dynamically switching between the chi-squared test, when all expected frequencies are greater than 5, and Fisher’s exact test. *P* values were approximated by the respective tests and subsequently adjusted for multiple comparisons using the Benjamini–Hochberg method. Significantly enriched GO terms were identified with an adjusted *P*-value cutoff of 0.05.

### Estimation of cell-to-cell variability

Scran^93,94^ was used to measure the cell-to-cell variability for each tissue. Briefly, the pool-based size factors were estimated and used for log normalization. Total variance for each gene was computed using modelGeneVar and decomposed into technical and biological variance. The biological component of variance for each gene is defined as the residual from the trend and was used to estimate gene expression variability. The mean variability across all genes for each tissue of each sample was calculated and used for comparison.

### Comparative analysis with mammalian aging

We computed pseudo-bulk data from the scRNA-seq data to reduce the dropout events. Both the sample-level (all tissues) and tissue-level (for example, intestine, and muscle) pseudo-bulk data were computed using the AggregateExpression function in Seurat. We mapped planarian genes to corresponding one-to-one *Mus musculus* orthologs and filtered out genes with a low number of reads, keeping only genes with at least 10 UMI counts in at least 20% of the pseudo-bulk samples, which resulted in 2,470 detected mammalian orthologs. Filtered data were then passed to relative log expression normalization. Age-related gene expression changes for the whole animal and every individual cell type were assessed using a linear model in edgeR^95^ with the following model: log(expression) ~ log(age).

To identify pathways enriched for age-related transcriptomic changes in planarians, we performed functional GSEA^69^ on a pre-ranked list of genes based on their log-transformed *P* values, corrected by the sign of regulation, calculated as:

$$-\left({\mathrm{pv}}\right)\times \mathrm{sgn}\left({\mathrm{slope}}\right),$$

where ‘pv’ and ‘slope’ are the *P* value and age-associated slope of expression for a certain gene, respectively, obtained from the edgeR output, and ‘sgn’ is the signum function (equal to 1, −1 or 0 if the value is positive, negative or equal to 0, respectively). REACTOME, KEGG and HALLMARK ontologies from the Molecular Signature Database were utilized as gene sets for GSEA. The fgsea package in R was used to run the GSEA algorithm with 5,000 permutations. An adjusted *P*-value cutoff of 0.1 was applied to select statistically significant functions.

A similar analysis was performed for mammalian gene expression signatures of aging and lifespan-extending interventions^70,71^. The human signature was based on skin, brain and skeletal muscle data from people ranging from 1 to ~96 years old. Kidney and liver tissue-specific signatures were identified using mouse and rat data, and the brain signature was based on mouse, rat and human samples. The aging signatures included multi-tissue age-related expression changes in human, mouse, rat and rodents (mice and rats) as well as tissue-specific aging biomarkers in the liver, brain and kidney. Longevity signatures included biomarkers of individual lifespan-extending interventions in mice, such as caloric restriction, growth hormone deficiency and rapamycin, along with transcriptomic changes associated with maximum and median lifespan in mice.

### Bulk RNA-seq and data analysis

Total RNA was extracted using the Monarch total RNA Miniprep Kit (NEB, T2010S). Poly-A selection was used to construct the libraries using the NEBNext Ultra II Directional RNA Library Prep Kit (NEB, E7760L).

Libraries were sequenced with NovaSeq 6000. Reads were subjected to quality control with FastQC (version 0.11.8), processed with Trimmomatic (version 0.38), aligned with STAR (version 2.7.1a) and counted with HTSeq (version 0.11.2) to produce transcript-per-million^96^ count matrices. Pearson correlation coefficients were calculated using the R function cor() and visualized with pheatmap (version 1.0.12). Differential expression analysis was performed using DESeq2 (ref. ^97^). *P* values were obtained with a two-sided Wald test and adjusted with Benjamini–Hochberg correction. Significant DEGs were identified with the threshold of adjusted *P* value < 0.05 and |log_2_FC | >1. The anteroposterior axis gene expression data of asexual planarians are provided as Supplementary Information^72^.

### Motility measurements

An automated video tracking setup was used to record the movements of animals^98,99^. Video recordings were taken for 24 h and analyzed with the video analysis software DDrop^98,99^. Based on the tracking data, the cumulative and total distances tracked for each animal in 24 h were calculated by DDrop. The video recording setup and the animal chamber were likely stressful for the planarians given the prolonged 24-h recording. The animals could not recover and were dead at the end of the recording. Our motility assay is likely a combinatory test of both stress resistance and neuronal/muscle functions.

### Measurement of ROS

To dissociate single cells, animals were finely minced and tissues were immersed in 1 mg ml^−1^ collagenase I (Sigma-Aldrich) with gentle agitation until a homogeneous cell suspension was achieved. The cells were incubated with 5 µM of CellROX Green (Invitrogen), 0.2 µM of MitoView (Biotium) and 1 µg ml^−1^ of Hoechst 33342 at room temperature for 1 h. Fluorescence-activated cell sorting and fluorescence intensity quantification were performed on Discover S8 (BD), with propidium iodide added right before analysis. FlowJo (Tree Star) was used to analyze and visualize the data.

### Planarian telomere restriction fragment analysis

Genomic DNA was extracted from whole planarians using the Easy-DNA kit (Invitrogen; K180001). Around 2–3 μg of planaria genomic DNA was digested with frequent cutters, HinfI and RsaI, overnight at 37 °C. Digested DNA was run on a 10-cm-long, 0.7% agarose-1× TAE gel, along with a λ DNA-HindIII digest ladder (NEB; N3012S) at a constant voltage of 70 V for 3 h. The gel was denatured in 0.5 M NaOH for 20 min at room temperature with shaking and washed with water for 10 min. The denatured gel was transferred to a sheet of Whatman filter paper and dried on a gel dryer at 50 °C for 1.5 h. The dried gel was neutralized in 0.5 M Tris-HCL (pH 7.5) for 15 min at room temperature with shaking and washed in water for 10 min. After neutralization, the gel was prehybridized in hybridization solution (5× SSC buffer, 5× Denhardt’s Solution (Bioworld; 10750005-2), 10 mM Na_2_HPO_4_ and 1 M Na_2_H_2_P_2_O_7_) at 42 °C with rotation in a hybridization oven for 15 min. Then, it was hybridized with a 5′ ^32^P-labeled telomeric G probe (GGTTAG)_2_ overnight. After hybridization, the gel was washed twice with 2× SSC for 15 min and twice with 0.1× SSC/0.1% SDS for 10 min. The gel was then exposed to a Phosphorimager screen for 24–96 h, and telomere length was visualized on an Amersham Typhoon biomolecular imager.

### RNA interference

Genes were cloned with cDNA libraries prepared from RNA of sexual *S. mediterranea* with the GeneRacer Kit (Invitrogen). Double-stranded RNA was synthesized by in vitro transcription using T7 RNA polymerase, and treated with DNase and purified using the Monarch RNA Cleanup Kit (NEB). RNA-mediated interference food was prepared by adding double-stranded RNA to liver paste (100 mg μl^−1^)^100^. The control double-stranded RNA was generated from a GFP inserted in the pJC53.2 vector, a kind gift from R. Roberts-Galbraith. Planarians were fed RNA-mediated interference food or control food every 4 days.

### In situ HCR

HCR RNA FISH (Molecular Instruments) was carried out as previously described^30,101^. Briefly, the adult animals were treated with 10% *N*-acetylcysteine for 10–15 min and fixed in 4% paraformaldehyde for 20 min. After washes with PBSTx (1× PBS + 0.5% Triton X-100), the animals were treated with Proteinase K for 20 min at room temperature. Probes and hairpins were used at 16 nM and 60 nM respectively. Samples were mounted with Prolong Diamond (Thermo Fisher) and procured for 24 h before imaging.

### Microscopic image collection and quantification

Live animals or fragments were imaged using a Leica S9i microscope with the Leica Application Suite (LAS X). Fiji (version 2.9.0/1.54d)^102^ was used to measure the area of the whole body or head of worms. We considered body regions anterior of the posterior ends of auricles as the heads. Relative head size was calculated as (head area)/(whole-body area).

Confocal images were taken with a Nikon A1 inverted point scanning confocal microscope. The same settings were used for all images of an experiment. Fiji and Imaris (version 9.9.1) were used for image processing and quantification. Cell counting was performed manually in a double-blinded fashion. The detailed parameters of cell counting for all experiments are in Supplementary Table 13.

Specifically, for *SOSd*^hi^ cells, we took a *z*-stack from the dorsal surface of the animal to the inside of the animal until the image had crossed the dorsoventral boundaries of the animal (D–V boundaries). When the confocal section hits the D–V boundary, the DAPI staining should be in sharp focus for the animal edges. After hitting the D–V boundary, *SOSd*^hi^ cells belonging to dorsal or ventral sides can be differentiated. For quantification, *SOSd*^hi^ cells at the D–V boundary were used.

### Statistics and reproducibility

We used one-way ANOVA with post hoc Tukey HSD test, Welch’s two-sample Student’s *t*-test, chi-squared test, linear regression (lm) or mixed models (lme4) in our data analysis and presentation. Some datasets were tested using multiple statistical methods with consistent conclusions. Differential gene expression analysis in single cells was performed with the embedded statistical methods of Seurat. No data were excluded from the analyses. Animals in Fig. 2 were randomly selected. The investigators were double blinded in confocal image quantification. No statistical methods were used to predetermine sample sizes, but our sample sizes are similar to those reported in previous publications^56,66,77^.

### Reporting summary

Further information on research design is available in the Nature Portfolio Reporting Summary linked to this article.

## Supplementary information

Supplementary Information Supplementary Discussion, Figs. 1–9 and uncropped gel scans.

Reporting Summary

Supplementary Video 1 The video shows confocal *z*-stack imaging of a planarian head with *SOSd* expression visualized by HCR-FISH, from dorsal to ventral. Step depth, 2 µm. The video shows *SOS*d^hi^ distribution, relative to epidermis, and D–V boundary. The end of the video is the D–V boundary.

Supplementary Table 1 Summary of scRNA-seq data, including the following information of each sample: name, age, sequencing saturation, total captured and filter passed cell number, thresholds for cell quality control, and median detected UMIs and gene number.

Supplementary Table 2 List of marker genes for cell-type annotation of each subcluster.

Supplementary Table 3 Quantification of *tgs-1*^+^ and *tspan-1*^+^ cell ratio in sigma neoblasts and *tspan-1*^+^ cell ratio in muscle neoblasts based on the scRNA-seq data.

Supplementary Table 4 Quantification of *cpeb1*^+^ cell ratio in each scRNA-seq sample.

Supplementary Table 5 Cell ratio of *Tert*^+^ and *POU-P1*^+^ cells in each neoblast subtype at different expression levels in the dataset from Extended Data Fig. 6.

Supplementary Table 6 Top 30 marker genes of different neoblast subtypes from Extended data Fig. 7e. *P* value was obtained using a two-sided Wilcoxon rank-sum test and adjusted using Bonferroni correction.

Supplementary Table 7 List of PAGs in each tissue. *P* value was obtained using a two-sided Wilcoxon rank-sum test and adjusted using Bonferroni correction. PAGs were identified with the threshold of adjusted *P* value < 0.05 and |avg_log_2_FC| > 0.25.

Supplementary Table 8 Significantly enriched GO terms among PAGs in each tissue. Two-sided chi-squared test (when all expected frequencies are greater than 5) or Fisher’s exact test was performed. *P* value was adjusted with Benjamini–Hochberg correction. Adjusted *P* value < 0.05.

Supplementary Table 9 List of functions enriched by at least one planarian aging signature. GSEA permutation test. *P* value adjusted for multiple comparisons with Benjamini–Hochberg correction.

Supplementary Table 10 List of DEGs between 0Hpa and 6Hpa in different tissues based on a published scRNA-seq dataset (CRX495534–CRX495535). *P* value was obtained using a two-sided Wilcoxon rank-sum test and adjusted using Bonferroni correction. DEGs were identified with the thresholds of adjusted *P* value < 0.05 and |avg_log_2_FC | > 0.25.

Supplementary Table 11 Age and body size information of samples used in this study.

Supplementary Table 12 List of cell cycle markers used to predict the cell cycle state of cells.

Supplementary Table 13 The detailed parameters and cell counts for cell counting performed in this study.

Supplementary Table 14 Coding sequences of important genes in this study.

## Source data

Source Data Fig. 1 Statistical source data.

Source Data Fig. 2 Statistical source data.

Source Data Fig. 3 Statistical source data.

Source Data Fig. 4 Statistical source data.

Source Data Fig. 4 Unprocessed gels.

Source Data Fig. 5 Statistical source data.

Source Data Fig. 6 Statistical source data.

Source Data Fig. 7 Statistical source data.

Source Data Extended Data Fig. 1 Statistical source data.

Source Data Extended Data Fig. 2 Statistical source data.

Source Data Extended Data Fig. 5 Statistical source data.

Source Data Extended Data Fig. 8 Statistical source data.

Source Data Extended Data Fig. 10 Statistical source data.

## Data Availability

Sequences for genes SOSd, *UBAC1* and *CA10* are in Supplementary Table 14 and uploaded to GenBank (PQ860516–PQ860518). All Illumina sequencing data are available at the NCBI Sequence Read Archive (BioProject no. PRJNA974485). Swiss-Prot (https://www.uniprot.org/help/downloads/) was used for homology search and GO analysis. Accession numbers for published datasets reanalyzed in this work include CRX495534, CRX495535, GSM8280219–GSM8280222 and GSM4404045–GSM4404051. Source data are provided with this paper.

## Code availability

No custom codes were used in the analysis of sequencing data. Codes used in the stress–motility assay are publicly available at https://github.com/PletcherLab/Arena_R_Code/tree/main/Code/.
