# Supplementary material for: Regeneration leads to global tissue rejuvenation in aging sexual planarians
Source: Nat Aging. 2025 Apr 3;5(5):780–98. doi: 10.1038/s43587-025-00847-9 (PMC12092299; doi:10.1038/s43587-025-00847-9)

nature aging

Supplementary information https://doi.org/10.1038/s43587-025-00847-9

Regeneration leads to global tissue rejuvenation in aging sexual planarians

In the format provided by the authors and unedited

Supplementary Discussion
Es-neo stem cells ----- Page 2
Body Sizes and Cell proportion ----- Page 2
Regeneration versus rejuvenation ----- Page 3
References

Supplementary Figures ----- Page 4

Uncropped gel scans

Supplementary Discussion

Es-neo stem cells

The differences between Issigonis et al. dataset and our dataset (Dai et al.) are:

a. FACS sorting to remove dead cells, versus no FACS.
b. Cells from trunk (anterior) and tail (posterior), versus head.

Integrated analysis of Issigonis et al. 2024 PNAS with Dai et al. provided several advantages:

a. Integration increased total cell numbers from 104,617 (current dataset) to 149,008, which will increase resolution power.
b. Both datasets were generated from 10x genomics and from sexual planarians.
c. Issigonis et al. dataset can be treated as independent repeat of our dataset in two different planarian body regions (trunk and tail), which are complementary to ours (head).
d. Integration excluded the concern that es-neo1/2 cells are dead cells.

Identification of es-neo in all three datasets (head, anterior and posterior) supports that es-neo stem cells are not dead cells and can be consistently profiled with single cell sequencing.

Higher mitochondria content or ribosome genes were sometimes used to infer damaged cells. However, mitochondria and ribosome also play important roles in stem cell biology[1-3]. If we consider high mitochondria as a damaged cell signature, it is not pervasive in our dataset as only epi_3 shared such a signature. This high specificity is hard to explain. In addition, to attribute both es-neo1 and es-neo2 to damaged cells is not sufficient to explain other observations: 1) How do es-neo1 and es-neo2 show complimentary gene expression patterns? 2) The stochasticity observed in es-neo1 and es-neo2 gene expression is shared by nu-neoblast which expresses ston-2 and tcf-1 more specifically and was extensively studied[4]. 3) Why does neoblast (stem cell) cluster have extensive "damaged cells" but not differentiated tissues? 4) The logical link between *Tert*^{high} cells in HCR-FISH and es-neo relies on the computational quantification of *Tert* expression in es-neo. If higher *Tert* expression in es-neo is created by current state of analysis algorithms (e.g., data transformation, normalization, integration), why high *Tert* was specific to a few stem cell clusters (e.g., es-neo1, es-neo2, nu)?

Even though the exact nature of es-neo clusters remains unclear, we present our best effort of annotation and discussion in this study. Future validation and study of es-neo stem cells is favored, potentially with more advanced technology or analytic algorithms.

Body Sizes and Cell proportion

It is worth mentioning that specific loss of neurons and muscle, but not stem cells, in older planarians, indicates that the cellular changes are due to differences in age, instead of size. Our data is distinct from the allometry associated cell proportion changes in a recent report[5], that involved an average of 6.45-fold differences in body sizes, and significant increase in frequencies of neoblasts, parenchymal, epidermal, phagocytes and goblet/basal cells, but decrease in frequencies of neurons, in larger animals. The sizes of animals used in our aging studies were

comparable, and documented in most experiments, or at least visually confirmed to be comparable before assays.

Regeneration versus rejuvenation

Is regeneration equivalent to rejuvenation? We remain puzzled that regeneration was not able to reverse cell-to-cell variability in epidermal and cathepsin cells, while in intestine, cell-to-cell variability was rejuvenated (**Fig. 5b**). In addition, regeneration only reversed 60-80% of the transcriptome in neural, intestine, muscle, epidermal, protonephridia and cathepsin tissues in older planarians. While we can attribute this to potential technical effect and animal variability, the minimal rejuvenation of the upregulated genes (~10%) in neoblast and secretory tissues suggest tissue specific regulatory mechanisms and that regeneration is not equivalent to rejuvenation. However, these data do support regeneration can induce the effect of rejuvenation.

References

1 Sharifi, S., da Costa, H. F. R. & Bierhoff, H. The circuitry between ribosome biogenesis and translation in stem cell function and ageing. *Mech Ageing Dev* **189**, 111282 (2020). https://doi.org/10.1016/j.mad.2020.111282

2 Sanchez, C. G. *et al*. Regulation of Ribosome Biogenesis and Protein Synthesis Controls Germline Stem Cell Differentiation. *Cell Stem Cell* **18**, 276-290 (2016). https://doi.org/10.1016/j.stem.2015.11.004

3 Pan, X. *et al*. Mitochondrial dynamics govern whole-body regeneration through stem cell pluripotency and mitonuclear balance. *Nat Commun* **15**, 10681 (2024). https://doi.org/10.1038/s41467-024-54720-1

4 Brown, D. D. R., Molinaro, A. M. & Pearson, B. J. The planarian TCF/LEF factor Smed-tcf1 is required for the regeneration of dorsal-lateral neuronal subtypes. *Dev Biol* **433**, 374-383 (2018). https://doi.org/10.1016/j.ydbio.2017.08.024

5 Elena, E., Alberto, P.-P., Maria, D. C. & Jordi, S. Allometry of cell types in planarians by single cell transcriptomics. *bioRxiv*, 2023.2011.2001.565140 (2023). https://doi.org/10.1101/2023.11.01.565140

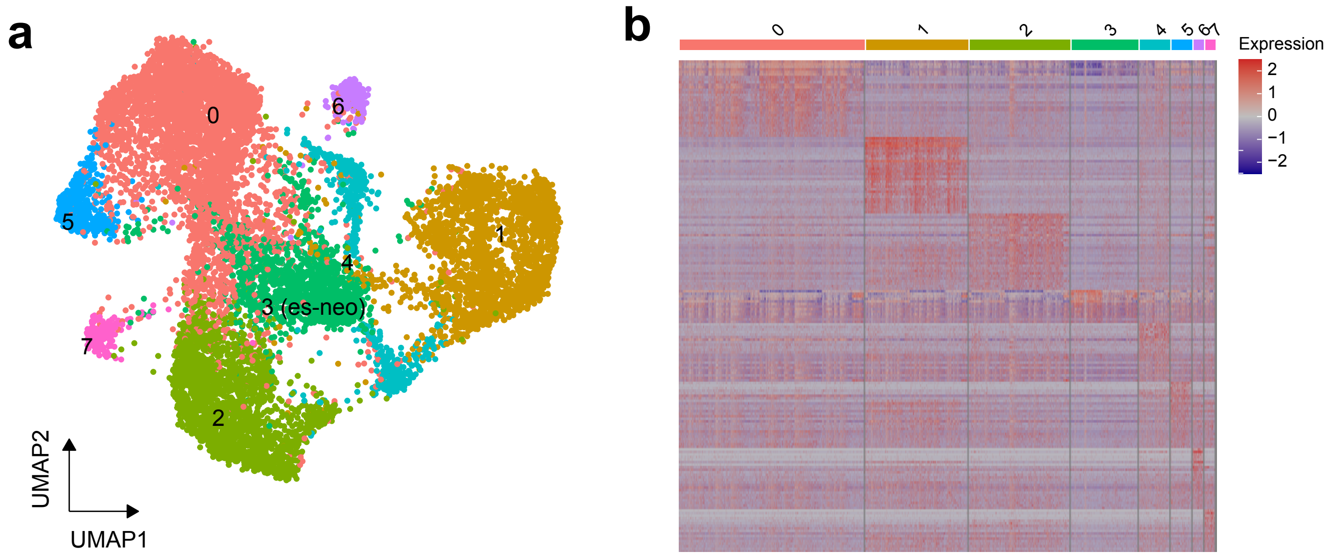

Supplementary Figure 1 Identification of es-neo in single-cell dataset of asexual planarians (a) UMAP projection of neoblast population from Benham-Pyle et al. 20218 of regenerating asexual planarian. (b) Heatmap of top 30 cluster markers of the neoblast populations from (a). Cluster 3 (stem cells-48) share similarities in gene expression patterns with es-neo cells.

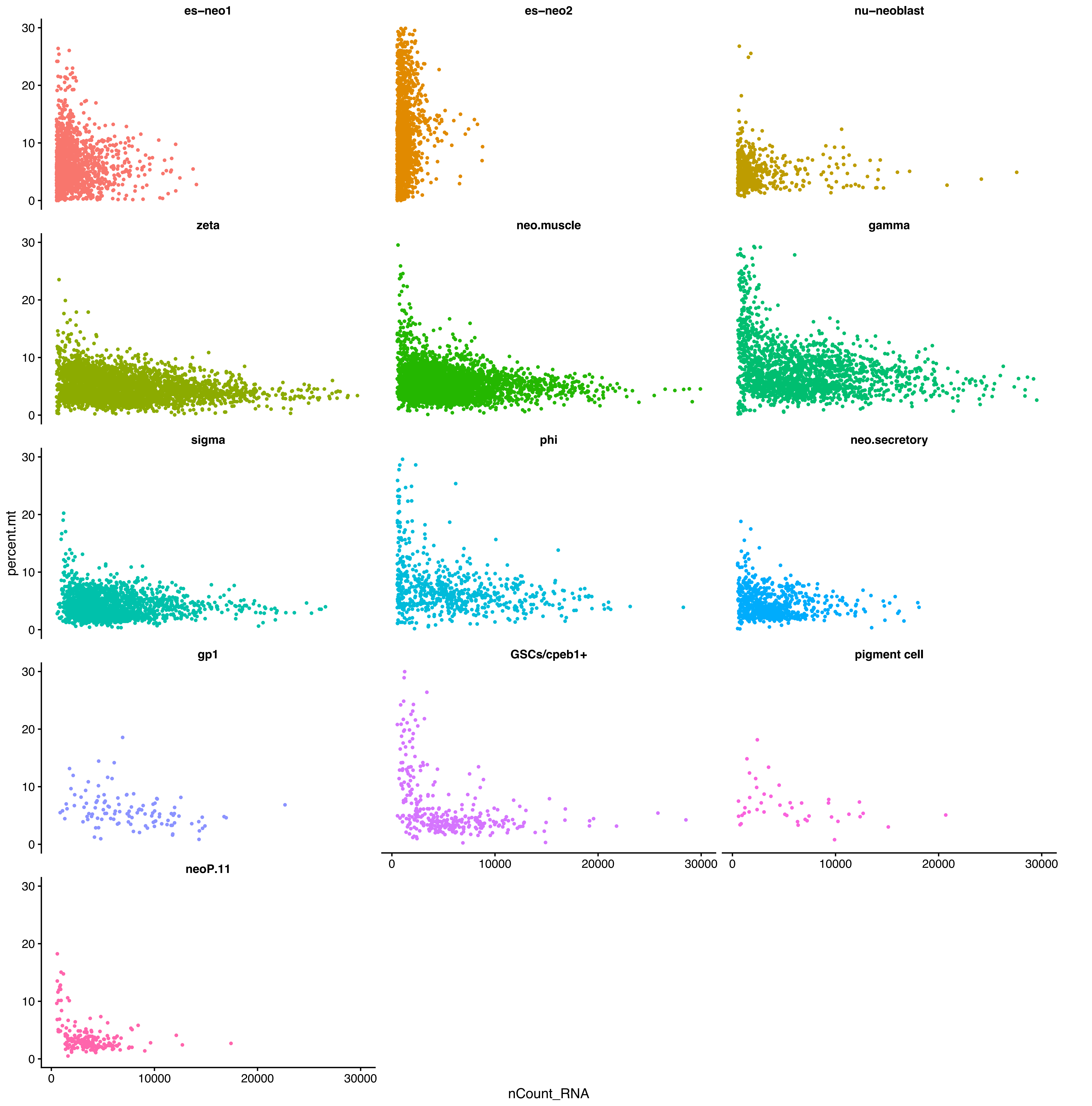

Supplementary Figure 2 Characterizing mitochondria and gene content
Scatter plot of neoblast subclusters showing detected transcript number (x-axis) and percentage of mitochondrial gene counts (y-axis) per cell.

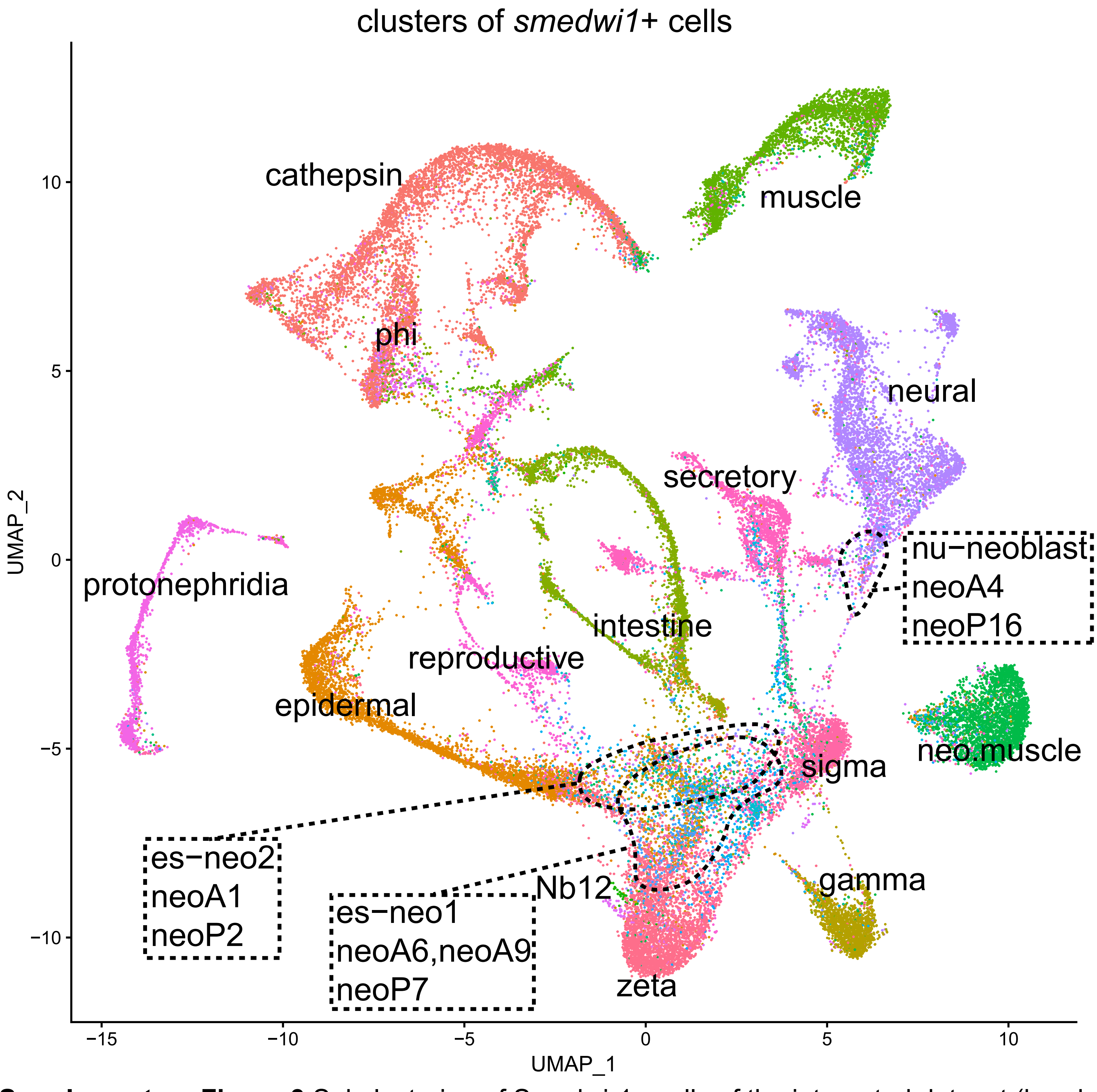

Supplementary Figure 3 Subclustering of Smedwi-1+ cells of the integrated dataset (head: current study, anterior: Issigonis et al., and posterior: Issigonis et al.).
Smedwi-1+ cells were extracted from the integrated dataset for subclustering analysis. Es-neo1, es-neo2, neoA.6, neoA.9, neoP.7, neoA.1 and neoP.2 co-localize in the juncture of multiple neoblast clusters (i.e., zeta, sima, epidermal, reproductive, secretory, neural etc.). Nu-neoblast are highlighted.

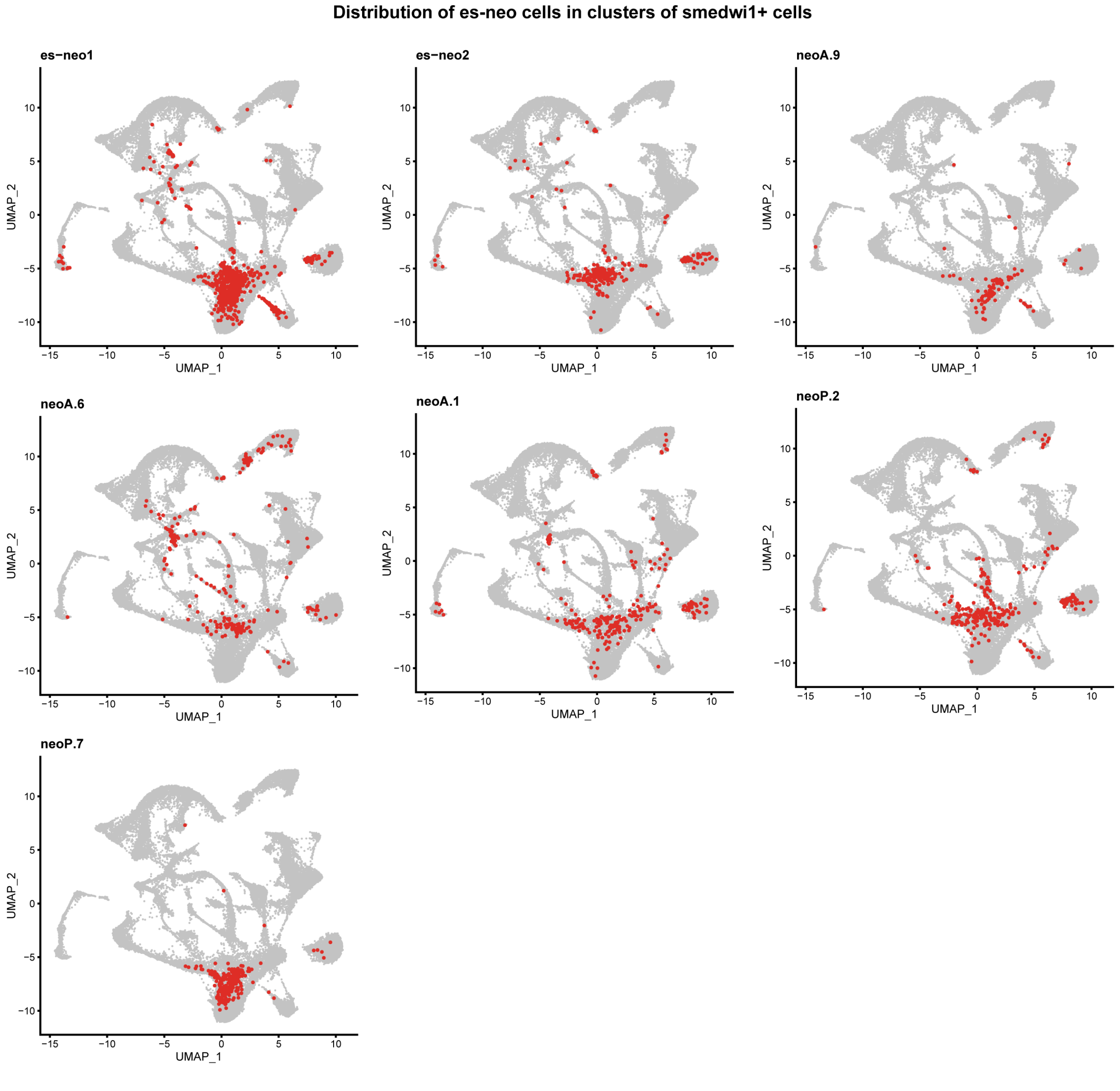

Supplementary Figure 4 Distribution of es-neo cells in subclusters of Smedwi-1+ cells in the integrated dataset.

Es-neo1 and es-neo2 are from head (current study). neoA.6, neoA.9 and neoA.1 are from anterior dataset (Issigonis et al.). neoP.2 and neoP.7 are from posterior (Issigonis et al.). All highlighted cell populations (red dots) share connections with other neoblast subclusters.

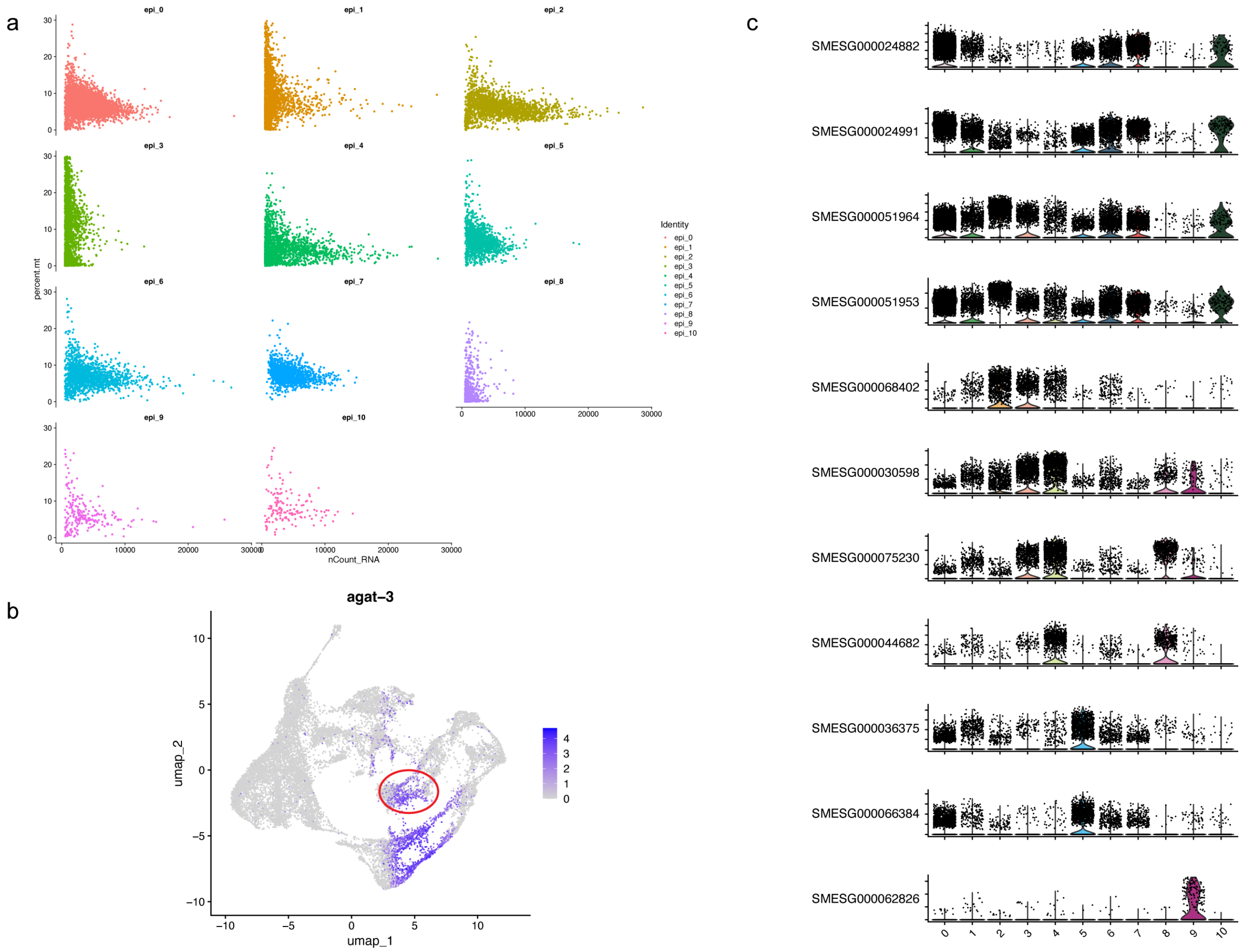

Supplementary Figure 5 Characterization of an epidermal subcluster, epi_3
(a) Scatter plot of epidermal subclusters showing detected transcript number (x-axis) and percentage of mitochondrial gene counts (y-axis) per cell. (b) Feature plot showing expression of agat-3 in epi_3 (red circle) cell types. (c) Violin plot showing expression of epidermal enriched genes in epidermal subclusters. Epi_3 (3 on the x-axis) does not express epidermal genes like es-neo1 and es-neo2 cells expressing neoblast genes.

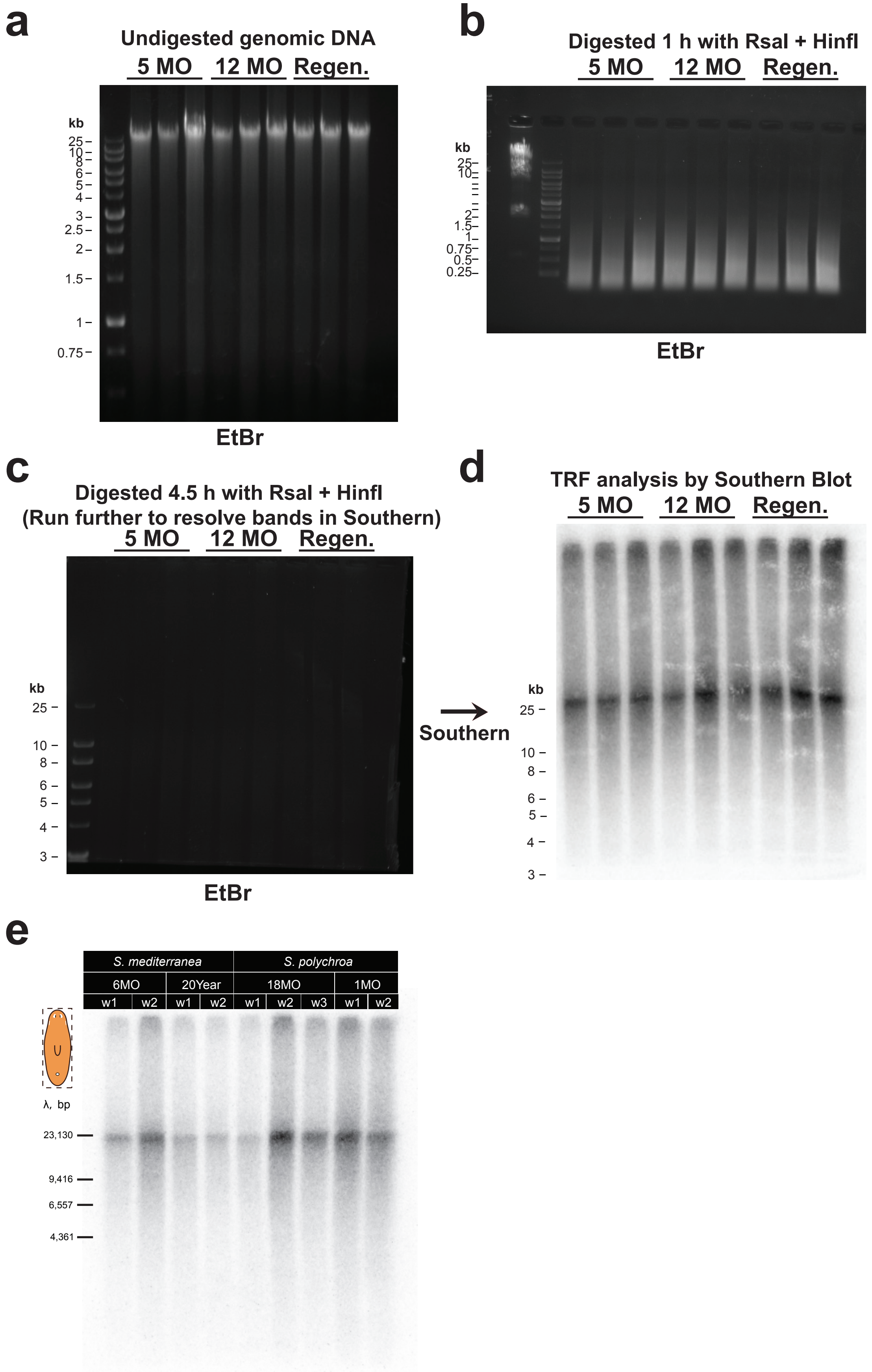

Supplementary Figure 6 TRF assay quality control and additional data on telomere
(a) Undigested genomic DNA with EtBr staining. (b) Genomic DNA with 1-hour RsaI and HinfI digestion. (c) Genomic DNA with 4.5-hour RsaI and HinfI digestion. (d) Southern blot of genomic DNA with 4.5-hour RsaI and HinfI digestion. (e) Telomere length measured by TRF analysis at different ages in different species. In all panels, one lane corresponds to DNA from one worm. MO: months. Regen: regenerated animals from 32MO animals.

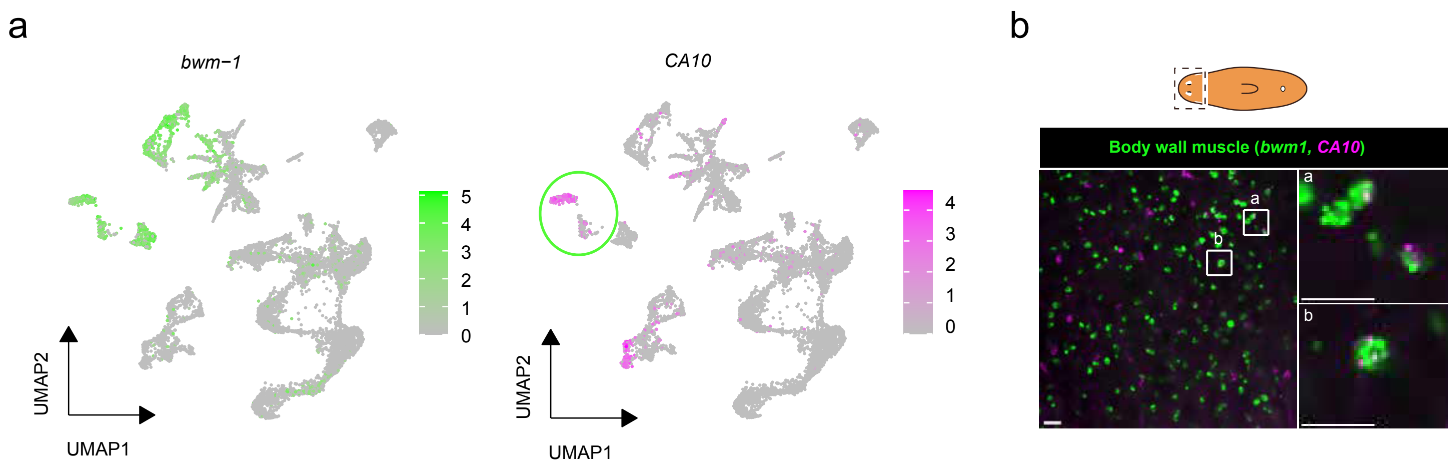

Supplementary Figure 7 Expression of muscle cell markers bwm-1 and CA10
(a) Feature plots to show that combined expression of bwm-1 and CA10 can specifically identify CA10+ body wall muscle. (b) HCR-FISH images showing expression patterns of body-wall muscle marker bwm-1 (green) and CA10 (magenta). Representative images of n = 3 individuals. Scale bar: 20 µm.

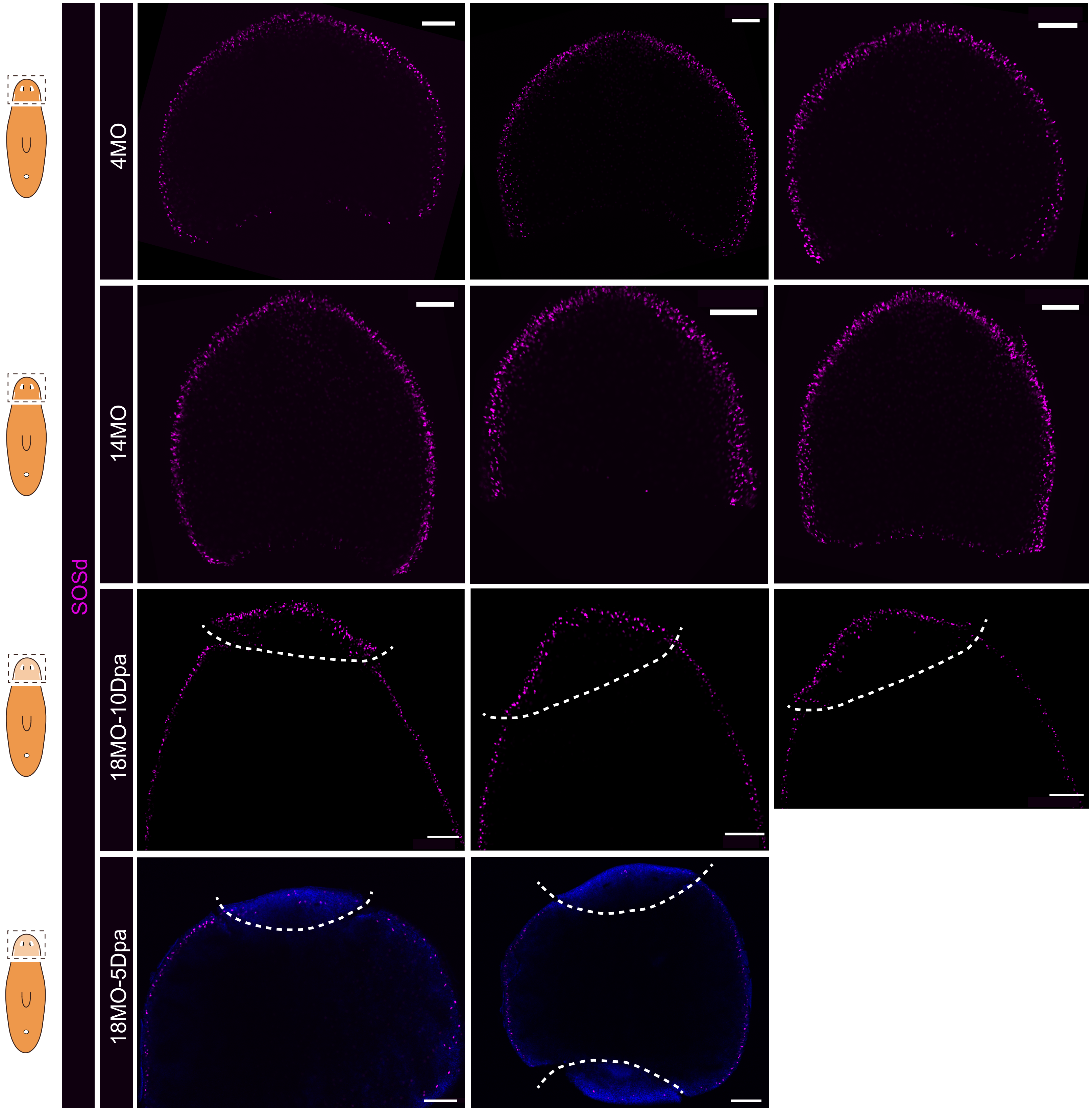

Supplementary Figure 8 HCR-FISH images of gene SOSd in planarian heads.
From top to bottom: young heads of 4 MO animals, aged heads of 14 MO animals, regenerating heads of 18MO animals 10 days post-amputation and 5 days post-amputation. Representative images of n ≥ 4 individuals from each condition. Scale bars = 200 µm. White dashed line: amputation plane. Single confocal sections (2 µm) representing the Dorsal-Ventral boundaries were shown.

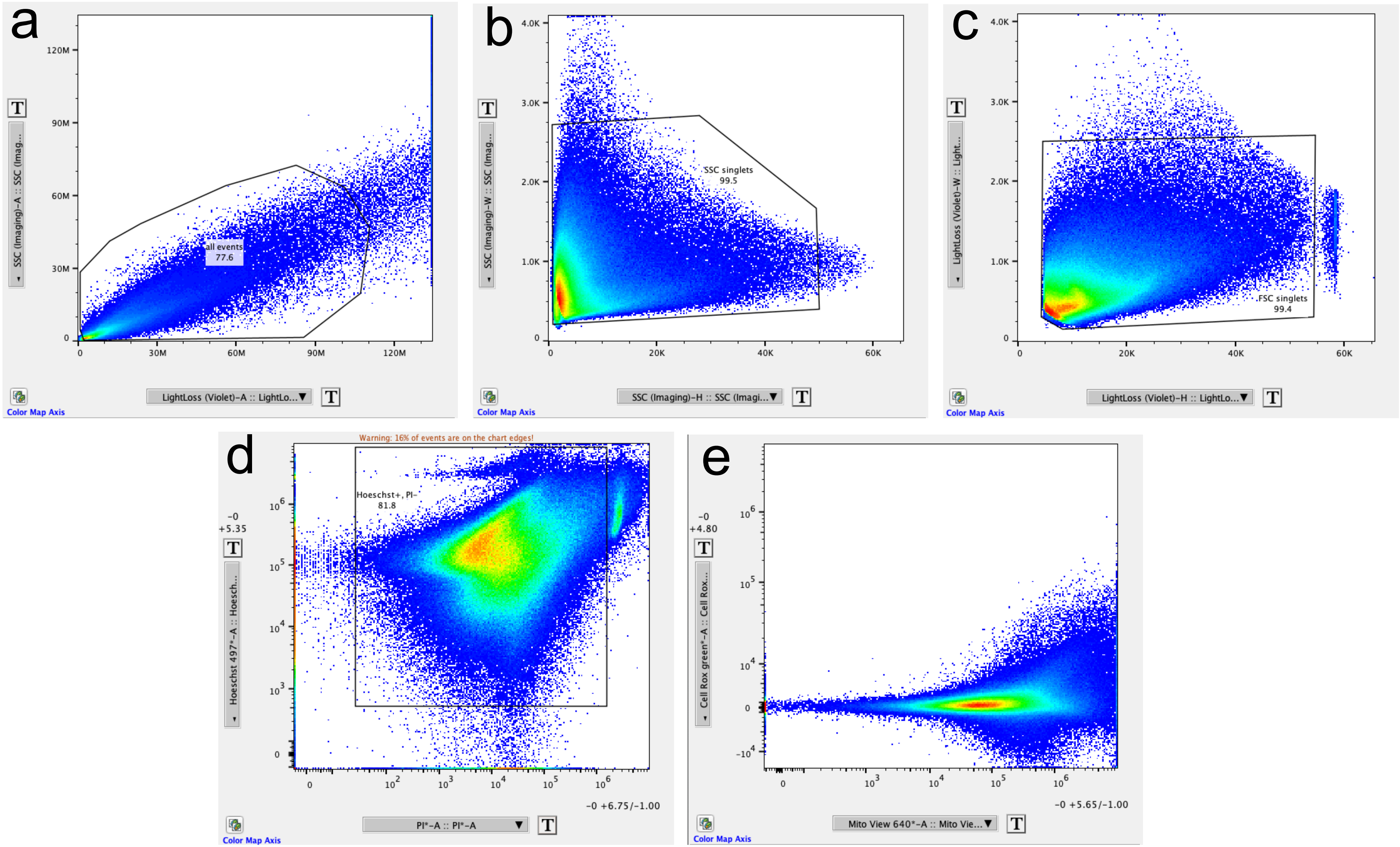

Supplementary Figure 9 Gating strategy for flow cytometry analysis in FlowJo
(a) Finding all cellular events with LightLoss Violet-A and SSC Imaging-A. (b) Selecting singlets with SSC Imaging-H and SSC Imaging-W. (c) Selecting singlets with LightLoss Violet-H and LightLoss Violet-W. (d) Selecting live cells with Hoechst positive and PI negative populations. (e) Analyzing cell distributions with Mito View 640 and Cell Rox green.

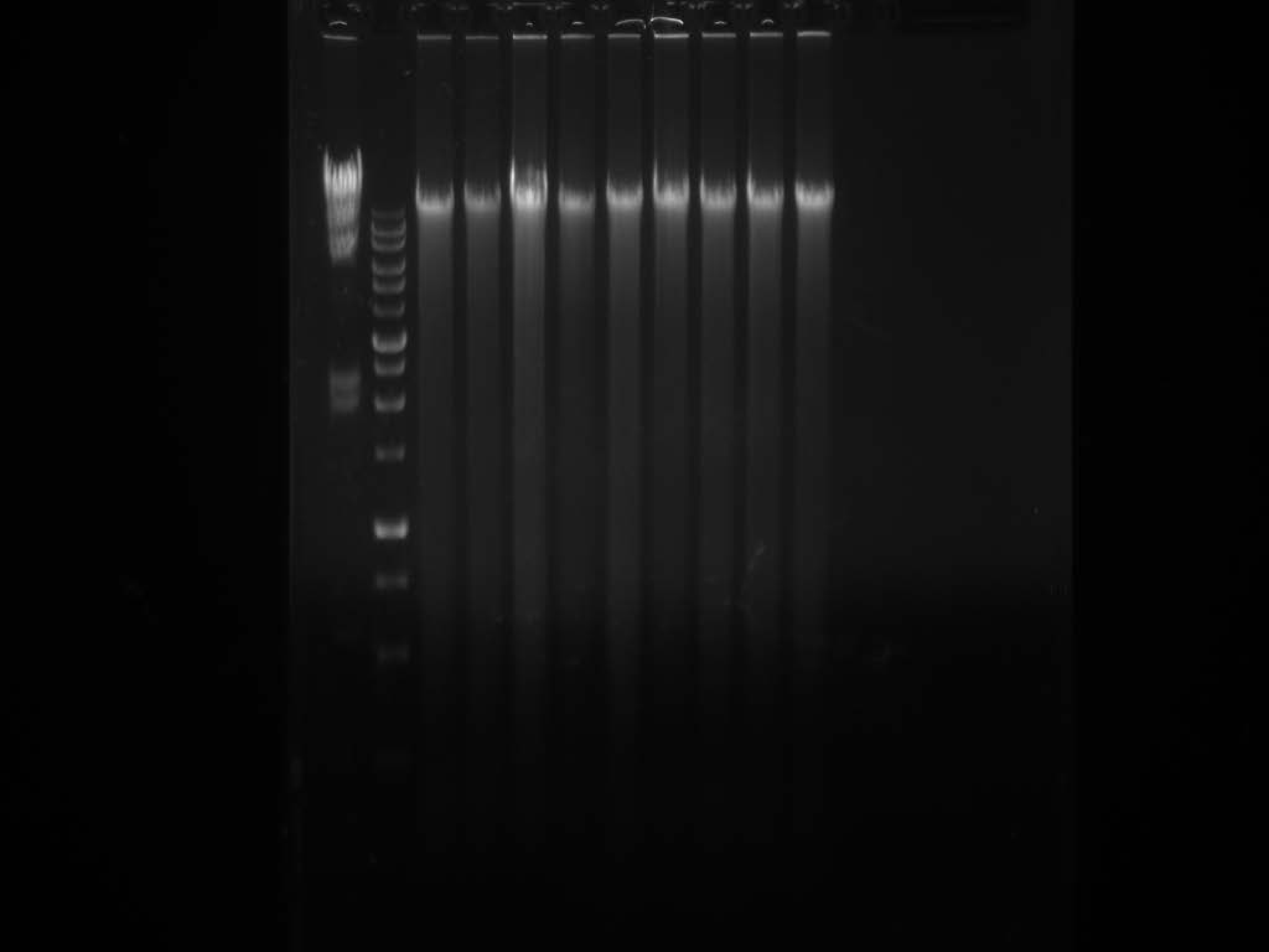

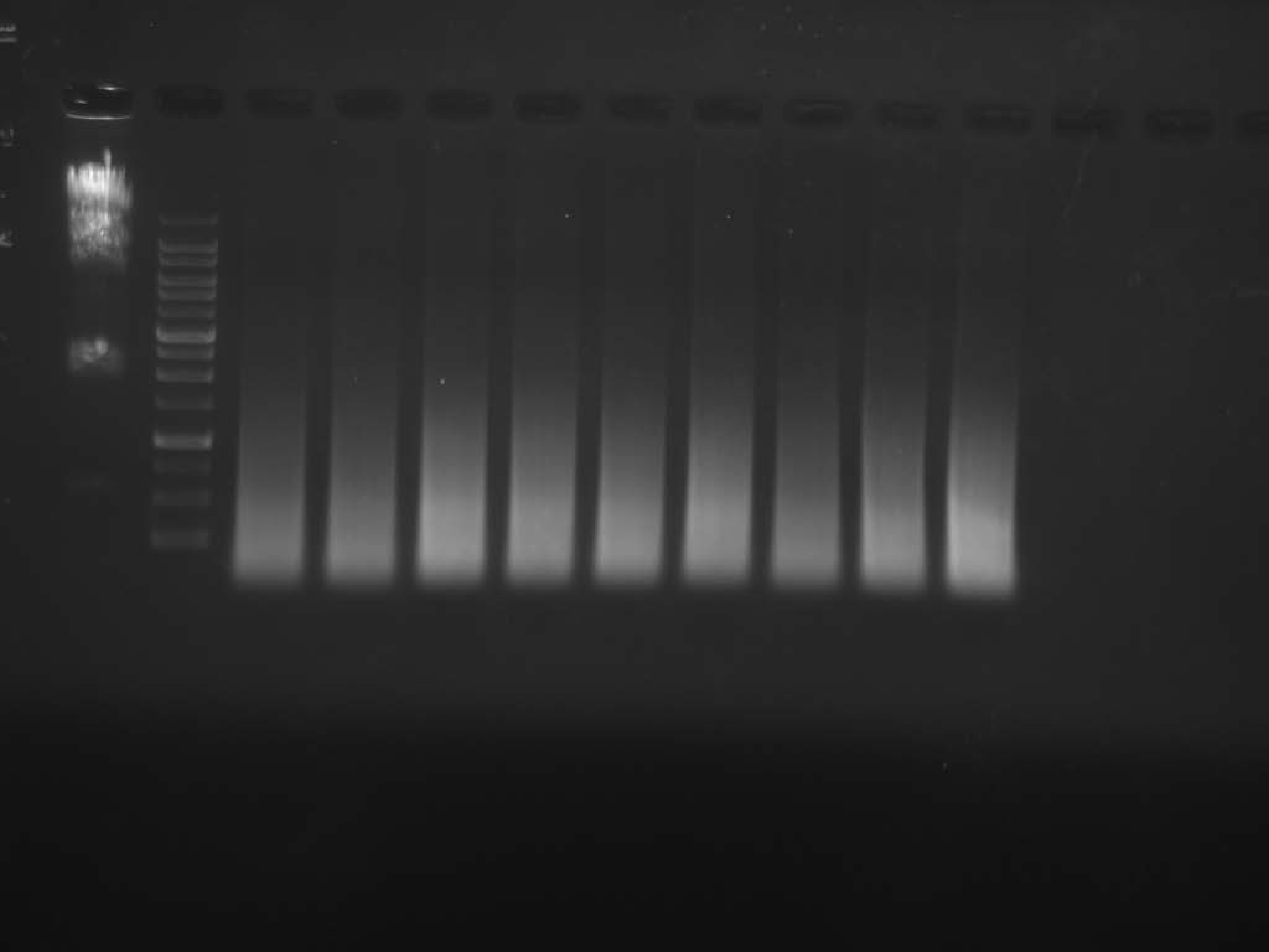

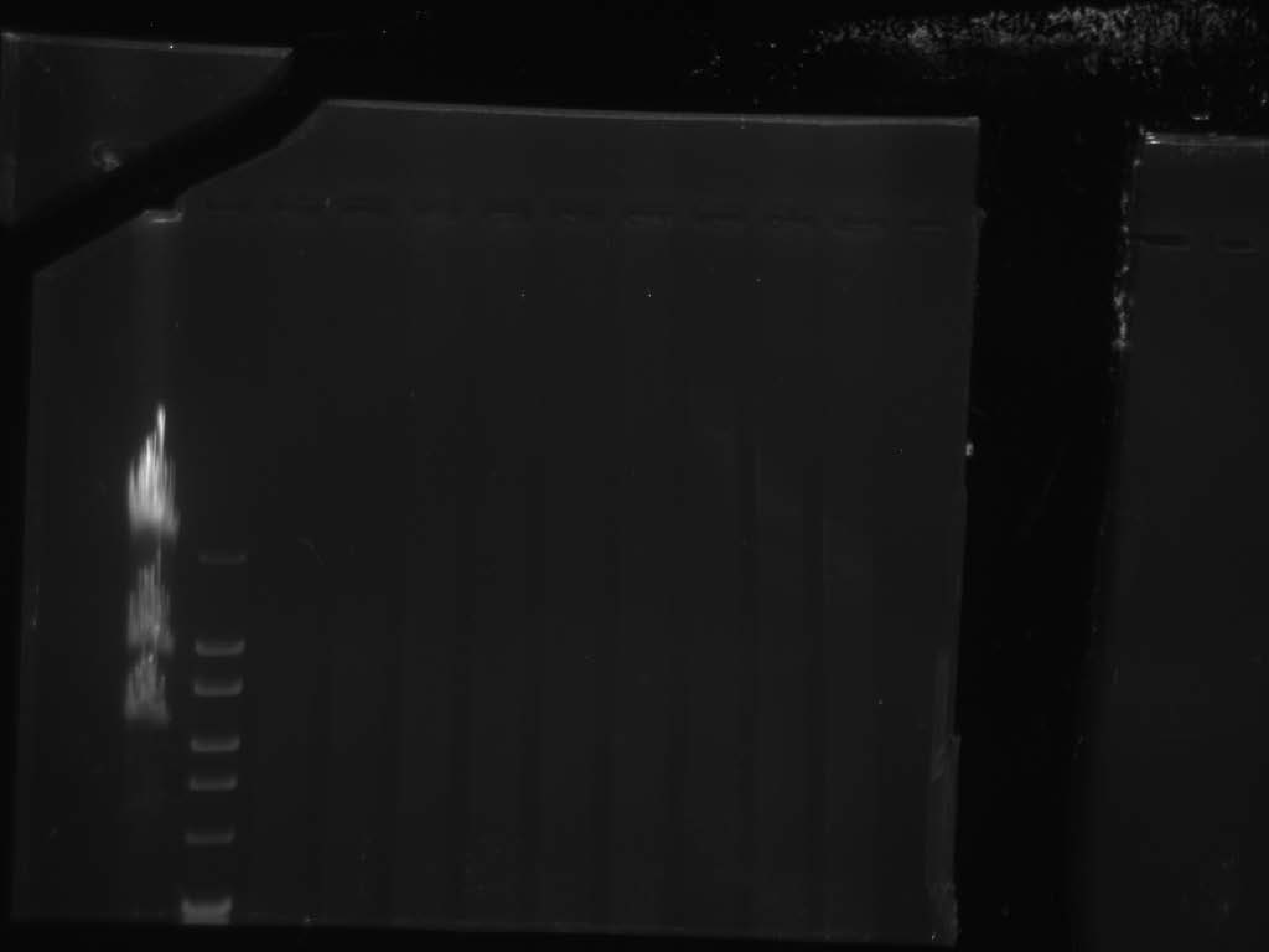

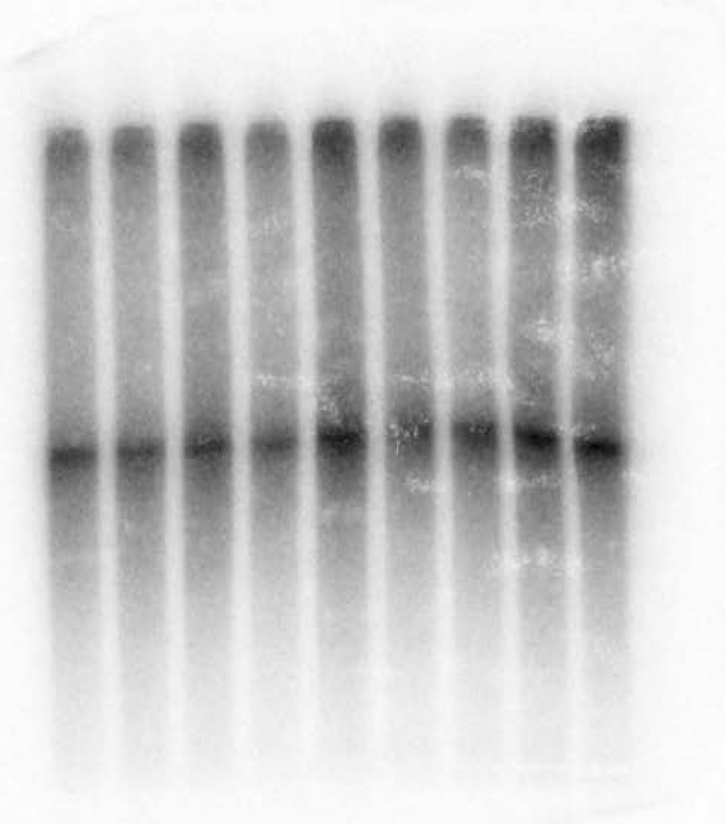

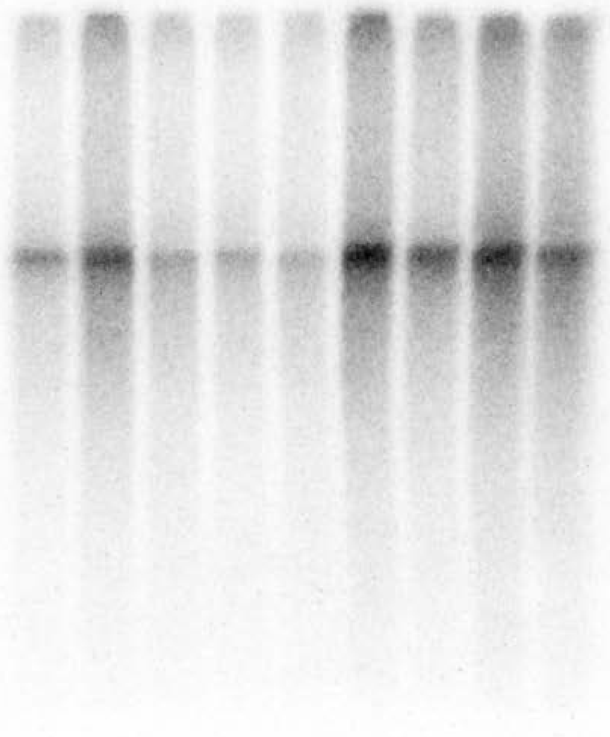

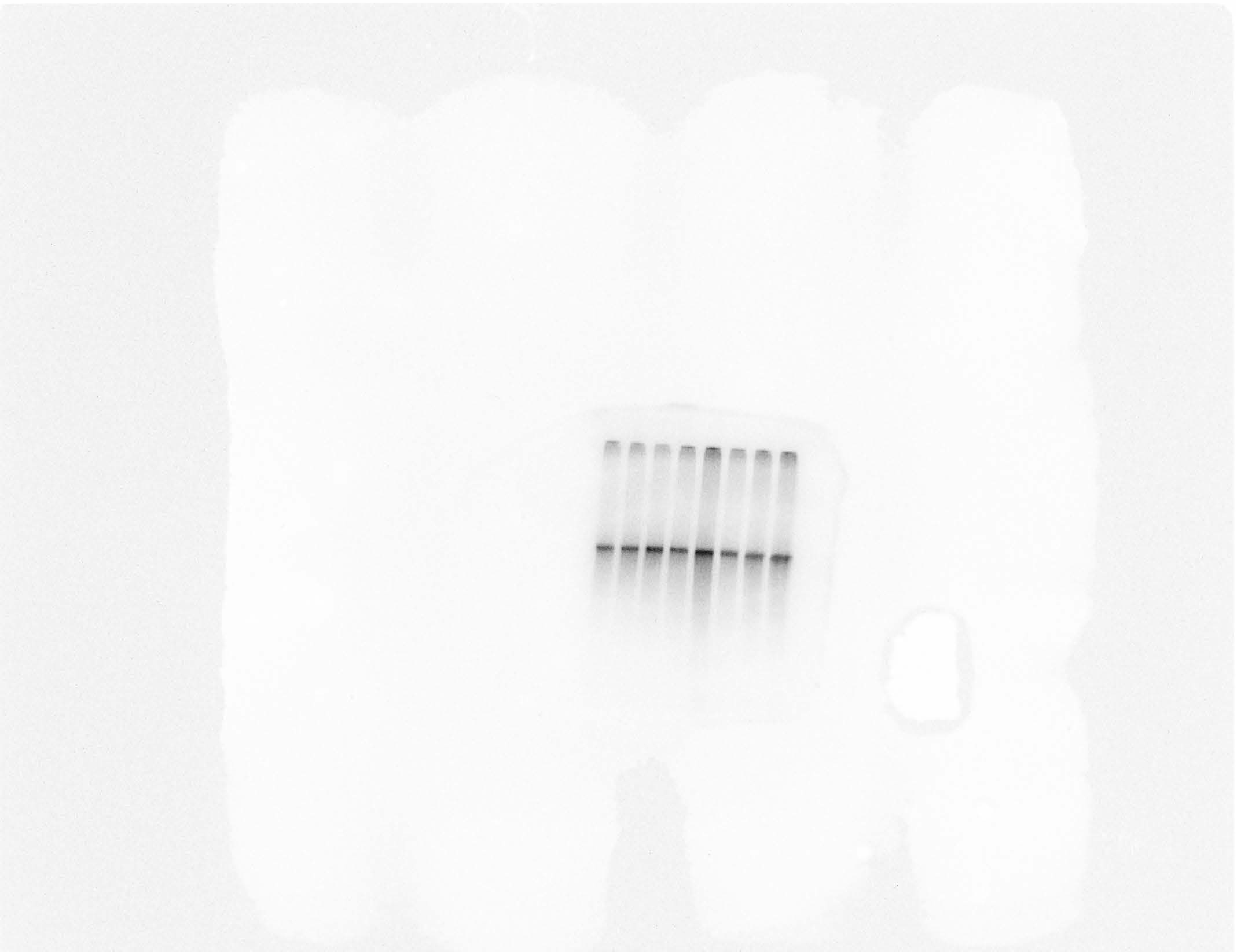

Supplement: Supplementary file 1 — Supplementary Discussion, Figs. 1–9 and uncropped gel scans. [file 43587_2025_847_MOESM1_ESM.pdf]
