# Supplementary material for: Regeneration leads to global tissue rejuvenation in aging sexual planarians
Source: Nat Aging. 2025 Apr 3;5(5):780–98. doi: 10.1038/s43587-025-00847-9 (PMC12092299; doi:10.1038/s43587-025-00847-9)
Supplement: Supplementary file 2 — Reporting Summary [file 43587_2025_847_MOESM2_ESM.pdf]

nature portfolio

Corresponding author(s): Longhua Guo

Last updated by author(s): Jan 21, 2025

Reporting Summary

Nature Portfolio wishes to improve the reproducibility of the work that we publish. This form provides structure for consistency and transparency in reporting. For further information on Nature Portfolio policies, see our Editorial Policies and the Editorial Policy Checklist.

Statistics

For all statistical analyses, confirm that the following items are present in the figure legend, table legend, main text, or Methods section.

n/a	Confirmed	
☐	☒	The exact sample size (*n*) for each experimental group/condition, given as a discrete number and unit of measurement
☐	☒	A statement on whether measurements were taken from distinct samples or whether the same sample was measured repeatedly
☐	☒	The statistical test(s) used AND whether they are one- or two-sided<br>*Only common tests should be described solely by name; describe more complex techniques in the Methods section.*
☐	☒	A description of all covariates tested
☐	☒	A description of any assumptions or corrections, such as tests of normality and adjustment for multiple comparisons
☐	☒	A full description of the statistical parameters including central tendency (e.g. means) or other basic estimates (e.g. regression coefficient) AND variation (e.g. standard deviation) or associated estimates of uncertainty (e.g. confidence intervals)
☐	☒	For null hypothesis testing, the test statistic (e.g. F, t, r) with confidence intervals, effect sizes, degrees of freedom and P value noted<br>*Give P values as exact values whenever suitable.*
☒	☐	For Bayesian analysis, information on the choice of priors and Markov chain Monte Carlo settings
☒	☐	For hierarchical and complex designs, identification of the appropriate level for tests and full reporting of outcomes
☒	☐	Estimates of effect sizes (e.g. Cohen's d, Pearson's r), indicating how they were calculated

Our web collection on statistics for biologists contains articles on many of the points above.

Software and code

Policy information about availability of computer code

Data collection	*Provide a description of all commercial, open source and custom code used to collect the data in this study, specifying the version used OR state that no software was used.*
Data analysis	CellRanger V6.0.1 was used to map the sequencing data to the genome and quantify the gene expression. DoubletFinder 2.0.3 was used in the process of data quality control. Seurat 4.2.1 was used to integrate, cluster, and visualize the single-cell gene expression data. R package Clustree was used to determine the best resolution for clustering. Velocyto 0.17.17, scVelo 0.2.5, and CellRank 1.5.1 were used to infer the lineage and trajectory. Cell cycle analysis and differential gene expression analysis were performed with Seurat built-in functions CellCycleScoring and FindMarkers. Gene ontology enrichment analysis was performed using EnrichPipeline 1.0.3. Cell-to-cell variability was estimated by employing the algorithm scran. The linear model in edgeR was used to assess age-related gene expression changes. GSEA was performed to identify pathways enriched for age-related transcriptomic changes in planarian and mammalian. Trimmomatic v0.38 was applied to cut adaptors and trim low-quality bases. STAR 2.7.1a was used to align the reads. The raw counts of aligned reads were obtained using the HTSeq (version 0.11.2). Pearson correlation coefficient was calculated using the R function cor() to evaluate the correlation of pairwise comparisons. Heatmap was generated using the pheatmap 1.0.12 R package. Differential gene expression analysis on bulk RNA-seq data was performed with DEseq2. FlowJo was used to analyzed the flow cytometry data. Images were processed using Fiji 2.9.0 and Imaris 9.9.1. DDrop (https://github.com/PletcherLab/Arena_R_Code/tree/main/Code) was used to quantify distances traveled in the stress-motility test.

For manuscripts utilizing custom algorithms or software that are central to the research but not yet described in published literature, software must be made available to editors and reviewers. We strongly encourage code deposition in a community repository (e.g. GitHub). See the Nature Portfolio guidelines for submitting code & software for further information.

Data

Policy information about availability of data

All manuscripts must include a data availability statement. This statement should provide the following information, where applicable:

- Accession codes, unique identifiers, or web links for publicly available datasets
- A description of any restrictions on data availability
- For clinical datasets or third party data, please ensure that the statement adheres to our policy

Sequences for genes SOSd, UBAC1, and CA10 are in Supplementary Table 14 and uploaded to GenBank (PQ860516-PQ860518). All Illumina sequencing data are available at NCBI SRA (Bioproject PRJNA974485). Swiss-Prot (https://www.uniprot.org/help/downloads) is used for homology search and GO analysis.

Research involving human participants, their data, or biological material

Policy information about studies with human participants or human data. See also policy information about sex, gender (identity/presentation), and sexual orientation and race, ethnicity and racism.

Reporting on sex and gender	na
Reporting on race, ethnicity, or other socially relevant groupings	na
Population characteristics	na
Recruitment	na
Ethics oversight	na

Note that full information on the approval of the study protocol must also be provided in the manuscript.

Field-specific reporting

Please select the one below that is the best fit for your research. If you are not sure, read the appropriate sections before making your selection.

☒ Life sciences ☐ Behavioural & social sciences ☐ Ecological, evolutionary & environmental sciences

For a reference copy of the document with all sections, see nature.com/documents/nr-reporting-summary-flat.pdf

Life sciences study design

All studies must disclose on these points even when the disclosure is negative.

Sample size	Sample size was determined based on similar studies, and standard practices that allow statistical testing. References for single cell analysis include 32669714 and 35613617 (PMID). References for animals used in FISH and RNAi design include 33473133 and 38889152 (PMID)
Data exclusions	No data was excluded, except those failed standard QC procedures in single cell RNAseq analysis.
Replication	To ensure robust reproducibility, age-related eye phenotypes have been observed in two specieses, S. mediterranea, and S. polychroa; Regenerate of new head experiment employed two ways for amputation, whole-head and half-head; The feeding behavior experiments were performed at least 4 times. The body-size changes after head amputation experiments were performed with 31 biological replicates. The fertility experiments were performed at least 4 times, involved two strains, LAF and S2Fn; The motility experiments were repeated 9 times; The oxidative stress experiments were performed in 6-8 biological replicates; The head size of 45-63 worms were measured in different age groups (63 individuals for young group, 45 individuals for old group). The Tert RNAi experiment was performed in 5-9 biological replicates. For scRNA-seq experiments, there were 6 biological replicates for the young condition, 7 biological replicates for the aged, and 3 biological replicates for the regenerated conditions; The bulk RNA-seq of tails were performed in 3 three biological replicates for each condition. The telomere length of two specieses, S. mediterranea, and S. polychroa, were measured and there are at least 2 replicates for each age group (S. mediterranea: 6 individuals for young group, 3 individuals for old group, and 3 for regenerated group; S. polychroa: 2 individuals for young group, 3 individuals for old group). The in situ HCR experiments were repeated at least 3 times.
Randomization	The animals used in this study were randomly selected from a group of certain ages, and then size-matched under different conditions
Blinding	Quantification of cells were carried out manually in a double-blinded fashion.

Reporting for specific materials, systems and methods

We require information from authors about some types of materials, experimental systems and methods used in many studies. Here, indicate whether each material, system or method listed is relevant to your study. If you are not sure if a list item applies to your research, read the appropriate section before selecting a response.

Materials & experimental systems

n/a	Involved in the study
☒	☐ Antibodies
☒	☐ Eukaryotic cell lines
☒	☐ Palaeontology and archaeology
☐	☒ Animals and other organisms
☒	☐ Clinical data
☒	☐ Dual use research of concern
☒	☐ Plants

Methods

n/a	Involved in the study
☒	☐ ChIP-seq
☐	☒ Flow cytometry
☒	☐ MRI-based neuroimaging

Animals and other research organisms

Policy information about studies involving animals; ARRIVE guidelines recommended for reporting animal research, and Sex and Gender in Research

Laboratory animals	Sexual lineage of Schmidtea mediterranea was used in this study. S. mediterranea of age between 1 month to 40 months were used for age-related eye phenotype characterization. S. mediterranea of 7 months, 8 months, 12 months, and 18 months of age were used for feeding behavior experiments. S. mediterranea of 6 months of age were used for repeated amputation and regeneration experiments. The fertility of two cohorts of S. mediterranea (LAF and S2Fn) were traced from the age of 200 days to 600 days. S. mediterranea of 4 months to 24 months of age were used for fertility rates comparison between young, old, and regenerated animals. S. mediterranea of 5 months, 17 months, and 35 months of age were used for the motility experiment. S. mediterranea of 6 months, 16 months, and 24 months were used for oxidative stress experiments. S. mediterranea of 4 months of age were used for Tert RNAi experiments. S. mediterranea of 5 months, 7 months, 12 months, 18 months, and 32 months were used for scRNA sequencing experiments. S. mediterranea of 4 months, 15 months, and 15 month-old-regenerated were used for bulk RNA-seq of tails. S. mediterranea of 1 month, 5 months, 6 months, 36 months, 20 years, and regenerated from 5-month-old worms were used for telomere restriction fragment analysis. S. mediterranea of 1 month, 4 months, 12 months, and 16 months were used for relative head size comparison. S. mediterranea of 5 months and 38 months were used for Tert/smedwi-1 HCR experiments. S. mediterranea of 4 months, 7 months, 14 months, 15 months, and 16 months were used for th HCR experiments. S. mediterranea of 4 months, 14 months, and 16 months were used for bwm1/CA10 HCR experiments. S. mediterranea of 21 days, 29 days, 34 days, 4 months, 7 months, 14 months, and 18 months of age were used for SOSd HCR experiments. S. mediterranea of 4 months and 14 months of age were used for UBAC1 HCR experiments. All details of animal ages and species were documented in the manuscript. Strain names were specified where needed. Majority of the visualized data were collected in strain LAF if not specified.
Wild animals	Schmidtea polychroa with abnormal eyes were underneath the rocks in creeks in Sardinia. They were transported in concealed 50ml tubes with planarian water. After observation, animals were maintained in the lab for experimentation. The age of the wild animals are unclear.
Reporting on sex	Both sexual S. mediterranea and S. polychroa were hermaphrodite.
Field-collected samples	Field-collected animals were maintained in 1x Montjuïch salts, in plastic containers, at 18°C, with once a week feeding of organic cow liver.
Ethics oversight	No ethical approval or guidance was required. Explain why not.

Note that full information on the approval of the study protocol must also be provided in the manuscript.

Flow Cytometry

Plots

Confirm that:

☒ The axis labels state the marker and fluorochrome used (e.g. CD4-FITC).

☒ The axis scales are clearly visible. Include numbers along axes only for bottom left plot of group (a 'group' is an analysis of identical markers).

☒ All plots are contour plots with outliers or pseudocolor plots.

☒ A numerical value for number of cells or percentage (with statistics) is provided.

Methodology

Sample preparation	To dissociate single cells, animals were finely minced and tissues were immersed in 1mg/mL collagenase I (Sigma-Aldrich, Munich, Germany) with gentle agitation until a homogenous cell suspension was achieved. Once the single cells were obtained, a specialized staining protocol was performed to label the cells for subsequent detection of ROS and mitochondrial activity. The cells were incubated with 5 µM of CellROX green (Invitrogen), 0.2 µM of MitoView (Biotium), and 1µg/mL of

Hoechst 33342 at room temperature for 1hour. Before analyzing the samples on the flow cytometer, propidium iodide was added to label dead cells.

Instrument	Fluorescence-activated cell sorting (FACS) analysis and fluorescence intensity quantification were performed on FACS Discover S8 (BD).
Software	FlowJo software (Three Star, Inc., Ashland, USA) was used to analyze the data.
Cell population abundance	SSC and FSC singlets were above 95%. Hoeschst positive and PI negative were 85-95%.
Gating strategy	gating was used in the following order: lightloss violet-A vs SSC imaging-A, SSC imaging-H vs SSC imaging W, lightloss Violet H vs Lightloss Violet W, PI vs Hoeschst, and Mito View vs Cell Rox. boundaries were defined based on general parameters for single cells, negative controls and single color controls

☒ Tick this box to confirm that a figure exemplifying the gating strategy is provided in the Supplementary Information.
