# Supplementary figures and images for: Regeneration leads to global tissue rejuvenation in aging sexual planarians
Source: Nat Aging. 2025 Apr 3;5(5):780–98. doi: 10.1038/s43587-025-00847-9 (PMC12092299; doi:10.1038/s43587-025-00847-9)

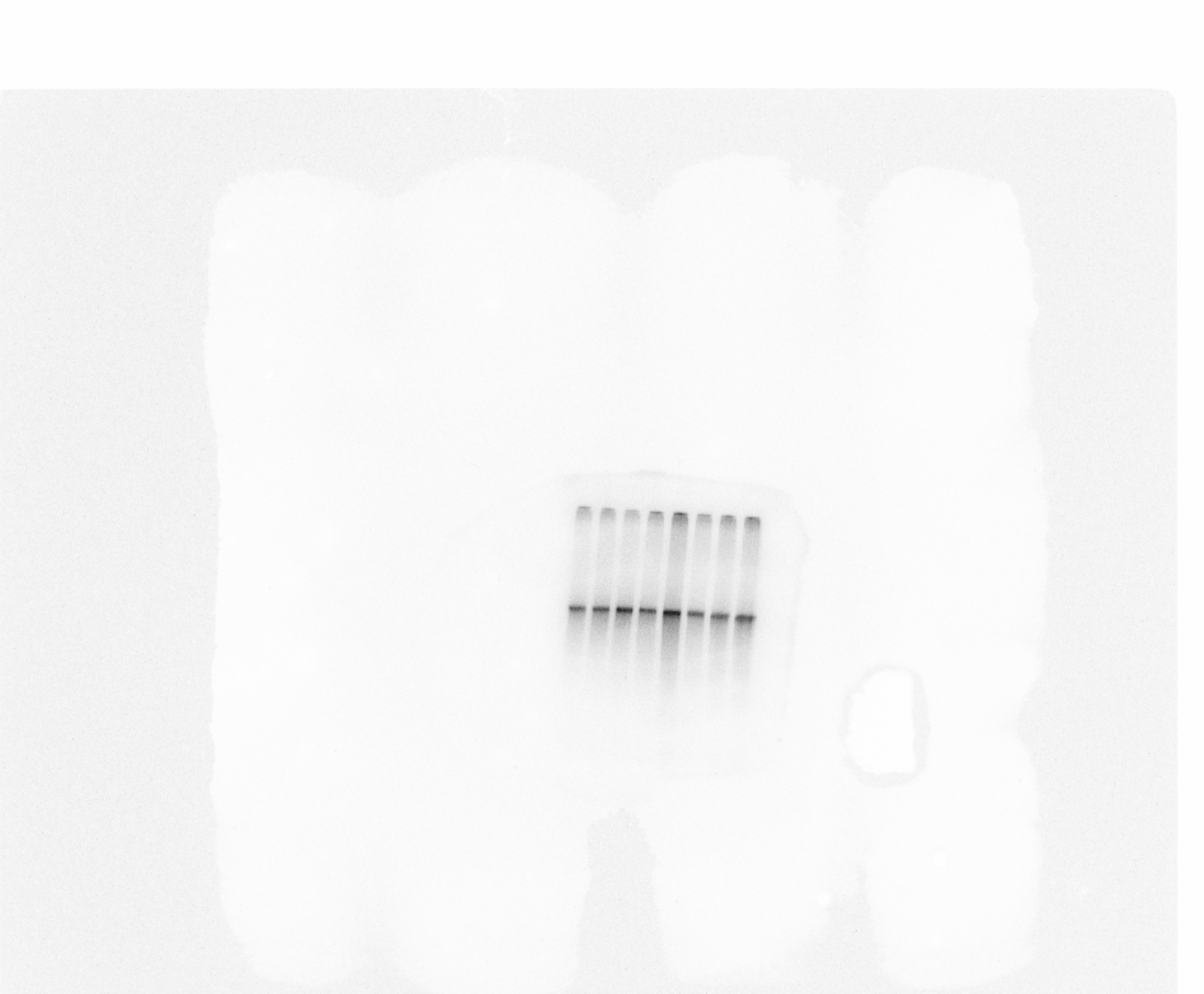

Supplement: Supplementary file 22 — Unprocessed gels. [file 43587_2025_847_MOESM22_ESM.tif]
